# Rates of increase of antibiotic resistance and ambient temperature in Europe: a cross-national analysis of 28 countries between 2000 and 2016

**DOI:** 10.2807/1560-7917.ES.2020.25.45.1900414

**Published:** 2020-11-12

**Authors:** Sarah F McGough, Derek R MacFadden, Mohammad W Hattab, Kåre Mølbak, Mauricio Santillana

**Affiliations:** 1 Harvard T.H. Chan School of Public Health, Harvard University, Boston, United States; 2 Computational Health Informatics Program, Boston Children’s Hospital, Boston, United States; 3 Division of Infectious Diseases, Department of Medicine, University of Toronto, Toronto, Canada; 4 Wyss Institute for Biologically Inspired Engineering, Harvard Medical School, Boston, United States; 5 Statens Serum Institut, Copenhagen, Denmark; 6 Institute of Veterinary and Animal Sciences, University of Copenhagen, Copenhagen, Denmark; 7 Department of Pediatrics, Harvard Medical School, Harvard University, Boston, United States

**Keywords:** antibiotic resistance, temperature, Europe

## Abstract

**Background:**

The rapid increase of bacterial antibiotic resistance could soon render our most effective method to address infections obsolete. Factors influencing pathogen resistance prevalence in human populations remain poorly described, though temperature is known to contribute to mechanisms of spread.

**Aim:**

To quantify the role of temperature, spatially and temporally, as a mechanistic modulator of transmission of antibiotic resistant microbes.

**Methods:**

An ecologic analysis was performed on country-level antibiotic resistance prevalence in three common bacterial pathogens across 28 European countries, collectively representing over 4 million tested isolates. Associations of minimum temperature and other predictors with change in antibiotic resistance rates over 17 years (2000–2016) were evaluated with multivariable models. The effects of predictors on the antibiotic resistance rate change across geographies were quantified.

**Results:**

During 2000–2016, for *Escherichia coli* and *Klebsiella pneumoniae*, European countries with 10°C warmer ambient minimum temperatures compared to others, experienced more rapid resistance increases across all antibiotic classes. Increases ranged between 0.33%/year (95% CI: 0.2 to 0.5) and 1.2%/year (95% CI: 0.4 to 1.9), even after accounting for recognised resistance drivers including antibiotic consumption and population density. For *Staphylococcus aureus* a decreasing relationship of −0.4%/year (95% CI:  −0.7 to 0.0) was found for meticillin resistance, reflecting widespread declines in meticillin-resistant *S. aureus* across Europe over the study period.

**Conclusion:**

We found evidence of a long-term effect of ambient minimum temperature on antibiotic resistance rate increases in Europe. Ambient temperature might considerably influence antibiotic resistance growth rates, and explain geographic differences observed in cross-sectional studies. Rising temperatures globally may hasten resistance spread, complicating mitigation efforts.

## Introduction

For almost a century, antibiotics have been our most effective way to treat bacterial infections, and antibiotics underpin enormous population health gains. However, soon after their initial introduction, bacterial pathogens demonstrated their propensity to acquire and propagate mechanisms to withstand the effects of these agents. Decades of unfettered use of antibiotics has been recognised as a main driver behind the selection and spread of resistant bacteria globally [1,2].

Antibiotic resistance poses one of the world’s greatest public health threats today, with the potential to render many existing classes of antibiotics ineffective in the near future [3,4]. To address this crisis, numerous national and international bodies have begun developing policy to control antibiotic use and funding research aimed at identifying and targeting drivers of resistance [3,5]. However, we still have an incomplete understanding of the factors beyond antibiotic consumption that influence the distribution and spread of antibiotic resistance in human populations [6-8].

Temperature is one of the strongest drivers of bacterial reproduction and can also modulate aspects of horizontal gene transfer through which resistance genes can be exchanged [9,10]. On a population level, ambient (air) temperature has been associated with rates of human carriage of pathogenic bacteria [11]. Ambient temperature additionally influences the intensity of human activities, such as food animal production, that could promote increased use of antibiotic agents [12]. Thus, directly or indirectly, temperature has the potential to modify the process of bacterial transmission across species, transfer of resistant mobile elements between bacteria, and selection of antibiotic resistant organisms at bacterial and human population scales. The effects of warming temperatures on a variety of infectious diseases globally have been identified by the World Health Organization; however, the impact of climate change on the distribution of antimicrobial resistance has been relatively ignored [13]. A recent ecologic study evaluated the distribution of antibiotic resistance in common bacterial pathogens across the United States (US) during the years 2013–2015, and found that antibiotic resistance prevalence was linked to local minimum ambient temperatures across geographies [14]. However, due to limited availability of historical data, the study could not demonstrate the temporal effects of climate on antibiotic resistance [14].

Here we use one of the most comprehensive antibiotic resistance databases in existence (from the European Antimicrobial Resistance Surveillance Network; EARS-Net) [15,16] to identify if the rates of change of antibiotic resistance across countries in Europe, over the years 2000–2016, may have been modulated by ambient temperature, and whether these findings may explain observed associations between temperature and antibiotic resistance across geographies.

## Methods

### Study design

We performed an ecologic analysis of the evolution of country-level antibiotic resistance prevalence in common bacterial pathogens over time across Europe, and evaluated associations with temperature and other predictors over a 17-year period (2000–2016). We built multivariate linear models of antibiotic resistance prevalence over time, for three common pathogens and up to four different subclasses of antibiotics. The study assessed 28 European countries: Austria, Belgium, Bulgaria, Croatia, Czech Republic, Denmark, Estonia, Finland, France, Germany, Greece, Hungary, Iceland, Ireland, Italy, Latvia, Lithuania, Luxembourg, the Netherlands, Norway, Poland, Portugal, Romania, Slovakia, Slovenia, Spain, Sweden, and the United Kingdom.

### Antibiotic resistance data

Antibiotic resistance data at the country level were collected as part of national surveillance for 28 countries across Europe, for three common bacterial Gram-positive and Gram-negative pathogens: *Escherichia coli* (Gram-negative)*, Klebsiella pneumoniae* (Gram-positive)*,* and *Staphylococcus aureus* (Gram-negative), representing approximately 3.4 million, 0.54 million, and 0.52 million tested isolates, respectively. These pathogens were chosen because they are the most common Gram-positive and Gram-negative pathogens that cause infections globally and align with a previously-published study that investigated these same three pathogens in the US [14]. 

Antibiotic resistance was identified as prevalence of resistance (% resistant) among reported isolates for a given year, country, and antibiotic subclass. Antibiotic susceptibility was evaluated, where available, for the following subclasses [14]: aminopenicillins (*E. coli*), 3rd generation cephalosporins (*E. coli* and *K. pneumoniae*), fluoroquinolones (*E. coli* and *K. pneumoniae*), aminoglycosides (*E. coli* and *K. pneumoniae*), and meticillin (*S. aureus*). Antibiotic resistance data originate from EARS-Net [16] and represent sterile source isolates from confirmed infections in the community and hospital (nosocomial). This publicly available database includes national annual human antibiotic resistance data across common bacterial pathogens for common drugs, for the majority of European countries over time periods dating back to 2000. These national data comprise one of the most comprehensive antibiotic resistance datasets in existence though constrain the analysis to the country level.

### Predictors and confounders of antibiotic resistance

#### Antibiotic consumption

*Antibiotic consumption* here refers to either sales data, reimbursement data, or both, depending upon the country. We do not distinguish between these two sources, rather referring to them both as ‘consumption’. To account for country level differences in antibiotic consumption, we used annual country level antibiotic consumption data from the European Surveillance of Antimicrobial Consumption Network (ESAC-Net) [17]. Specifically, the data employed included the number of defined daily doses (DDD) per 1,000 inhabitants per day, from combined inpatient and outpatient sources (where available) for all major Anatomical Therapeutic Chemical (ATC) Classification System antibiotic classes. These data from The European Surveillance System – TESSy, were provided by Austria, Belgium, Bulgaria, Croatia, Czech Republic, Denmark, Estonia, Finland, France, Germany, Greece, Hungary, Iceland, Ireland, Italy, Latvia, Lithuania, Luxembourg, Netherlands, Norway, Poland, Portugal, Romania, Slovakia, Slovenia, Spain, Sweden, and the United Kingdom, and released by the European Centre for Disease Control and Prevention (ECDC). For each country, the data were available for up to 17 years, from 2000 to 2016, and were reported by national bodies such as drug registers, ministries of health, and health insurance companies covering 100% of the population. Antibiotic consumption data were missing for some countries and years, and the number of countries with data increased over time. The following antibiotics were represented by the following ATC codes: penicillins – J01C (including meticillin – J01CF), fluoroquinolones – J01M, aminoglycosides – J01G, and 3rd generation cephalosporins – J01DD. We note that antibiotic use may also be correlated with other population-specific factors that could influence the likelihood of resistance (e.g. proportion of individuals in chronic care or institutionalised settings), and thus some of these unmeasured factors may be adjusted for, as a result of its inclusion in the analysis model.

#### Minimum ambient temperature

Our second predictor was ambient temperature. Given that *minimum temperature* has been identified as important when describing species survival [18,19] and was found to have meaningful associations with antibiotic resistance prevalence in a previous ecological study [14], we focused on extracting this attribute. Constrained by the spatial resolution of the antibiotic resistance data to a country-level analysis and noting that certain European countries possess large north–south latitudinal ranges or topographical variations that may limit human population settlements, we used three different approaches to calculate annual minimum temperature values for our study.

First, we calculated the country-level annual average minimum temperature using modelled and assimilated meteorological data, available at a native geographic resolution of 0.5° × 0.625° from the Modern-Era Retrospective analysis for Research and Applications, Version 2 (MERRA-2) [20]. The MERRA-2 data are publicly available through the Global Modeling and Assimilation Office (GMAO) at the National Aeronautics and Space Administration (NASA) Goddard Space Flight Center. MERRA-2 contains hourly information from 1980 through the present date, with no missing data and geographic coverage. This consistency over time and space makes MERRA-2 an ideal data source for this analysis. For this approach, we extracted the daily minimum temperature for the 17-year period (2000–2016) from MERRA-2 gridded rasters onto a European shapefile containing country-level attributes. We took the weighted mean of daily minimum temperature across grid cells spanning each country (with weights proportional to the fraction of the cell falling within the country border), and computed the annual mean, for each country, as the mean of daily mean minimum temperature over the calendar year. We refer to this value as ‘annual average minimum temperature’ or ‘minimum temperature’ for brevity. The shapefile was obtained from the Eurostat Geographic Information System of the Commission (GISCO) of the European Commission, and geodata extractions were performed with the aid of the ‘rgdal’ and ‘raster’ packages in R (Version 3.3.2) [21].

The other two approaches to calculate minimum temperatures were conceived to assess the sensitivity of our findings to our choice of climate data source and spatial resolution. For these two subsequent approaches, we used city-level temperature data obtained from (i) MERRA-2 data and (ii) local weather stations. For (i), daily minimum temperature from MERRA-2 were obtained from the single 0.5° × 0.625° grid cell covering the centroid of each capital city, and the annual mean for each country was computed over the calendar year. For (ii), we obtained annual average minimum temperature from the most complete weather station data corresponding to a capital or populous city, as provided by the European Climate Data & Assessment (ECD&A) project of the Royal Netherlands Meteorological Institute (KNMI). A total of 26 of the 28 countries had available weather station data, and the list of cities used for each country can be found in Table S1. It is important to note that using weather station data removes the theoretical modelling assumptions implicit in reanalysis data such as MERRA-2.

#### Population density

We computed the population density (persons/km^2^) for each country and year using annual population estimates obtained through Eurostat. 

#### Country

We included country as a predictor in order to capture country-level confounding effects, which inherently adjusts for all non-time varying predictors.

### Data analysis

We initially assessed the pairwise associations between relevant predictors, including annual average minimum temperature, population density and antibiotic consumption, and antibiotic resistance across countries, pathogens and antibiotic subclasses. Additionally, we plotted the relationship between minimum temperature and antibiotic resistance within levels of predictors (antibiotic consumption (median), and population density (tertile)) to assess for potential confounding and effect modifications. 

Log scales were used for antibiotic consumption given the large spread of values. To ease visualisation of antibiotic resistance across multiple different antibiotics, we centred the data about the mean and normalised by the standard deviation, by pathogen, antibiotic, and year. To further visualise temporal trends, we computed the slope representing the effect, by year, of minimum temperature on antibiotic resistance as well as the rate of change of antibiotic resistance over the 17-year period. These were done for each pathogen type and subclass of antibiotic.

To visualise the distribution of antibiotic resistance and minimum temperature across Europe, we generated choropleth maps of (i) normalised antibiotic resistance across the three pathogens and (ii) annual average minimum temperature, each summarised over the 17-year time series. To produce a summary measure of antibiotic resistance, we first computed the weighted average of antibiotic resistance for each year, normalised by pathogen and antibiotic subclass and weighted by the number of isolates tested for a given pathogen and antibiotic subclass. The simple average of these values across years was then used to visualise the average normalised antibiotic resistance per country between 2000 and 2016. We also mapped the annual average minimum temperature by country as the mean of annual minimum temperature over the 17 years. Maps and figures were generated using the ‘ggplot2’ package in R.

### Statistical analysis

Multivariate linear models were used to measure the association between minimum temperature (°C) and antibiotic resistance prevalence over time, adjusting for country, year, population density (persons/km^2^), antibiotic consumption (DDD per 1,000 inhabitants per day), and the interaction between time and minimum temperature. These models were replicated across each pathogen and antibiotic subclass. They had the structure presented in Equation 1, where *Tmin* is the minimum temperature in Celsius, *time* the calendar year, *Population Density* the population density (persons/km^2^), *Antibiotic Consumption* the antibiotic consumption (DDD/1,000 inhabitants/day), and *Country_j_* the dummy variable for the j^th^ country, where j = (28 − 1 = 27). *β_1–27_* represent the effect of these variables on antibiotic resistance. The linear model assumes a normal distribution of errors (*ε*).

$$\begin{array}{l} \left(1\right)\;Antibiotic\;resistance=\\ \beta_{0}+\beta_{1}Tmin+\beta_{2}time+\beta_{3}Tmin*time+\beta_{4}Population\;Density+\beta_{5}log(Antibiotic\;Consumption)+\sum_{j=1}^{27}\beta_{5+j}Country_{j}+\varepsilon ,\varepsilon ∼N(0,\sigma^{2}) \end{array}$$

The interaction term between time and minimum temperature was included to assess the extent to which temperature is associated with the change in antibiotic resistance over time. To quantify the association of the temporal changes of antibiotic resistance prevalence with minimum temperature, we calculated the time derivative of Equation 1:

$$\begin{array}{l} \left(2\right)\;\frac{d}{dt}Antibiotic\;resistance=\\ \beta_{2}+\beta_{1}\frac{d}{dt}Tmin+\beta_{3}\left(Tmin+time*\frac{d}{dt}Tmin\right)+\beta_{4}\frac{d}{dt}Population\;Density+\beta_{5}\frac{d}{dt}log(Antibiotic\;Consumption) \end{array}$$

Equation 2 captures how the rate of change of antibiotic resistance evolves in time for each pathogen and antibiotic subclass. To determine the contribution of each of the terms to rates of change of antibiotic resistance, we estimated their values by approximating the time derivatives (of *Tmin*, *Population Density*, and *Antibiotic Consumption*) by the observed changes from year to year in the data. We then calculated the distributions of values for each individual term across countries and years. Given that *β_2_* is constant across years and countries, we performed this step on the four rightmost terms of Equation 2.

We chose a multivariate linear model with random country specific intercepts over a more complicated hierarchical model given that it was parsimonious, methodologically robust, and with a constrained number of degrees of freedom (a hierarchical model would greatly increase the number of predictors in a limited sample size of 28 countries and 17 years).

## Ethical statement

All relevant ethical guidelines were followed to conduct this study; due to the use of publicly available, anonymised and aggregated data, this study did not require an institutional review board (IRB) and/or ethics committee approval.

## Results

### Antibiotic resistance temporal trends across Europe

Antibiotic resistance in *E. coli* and *K. pneumoniae* has increased over time for most countries in Europe (Supplementary Figures S1, S2), while *S. aureus* resistance to meticillin has generally decreased over time (Figure S3). In the same time period, however, country-specific temporal trends in two potential predictors of resistance, minimum temperature and antibiotic consumption, show no clear temporal tendencies in Figures S4 and S5. Countries have generally experienced steady increases or decreases in population density (Figure S6).

**Figure 1:**
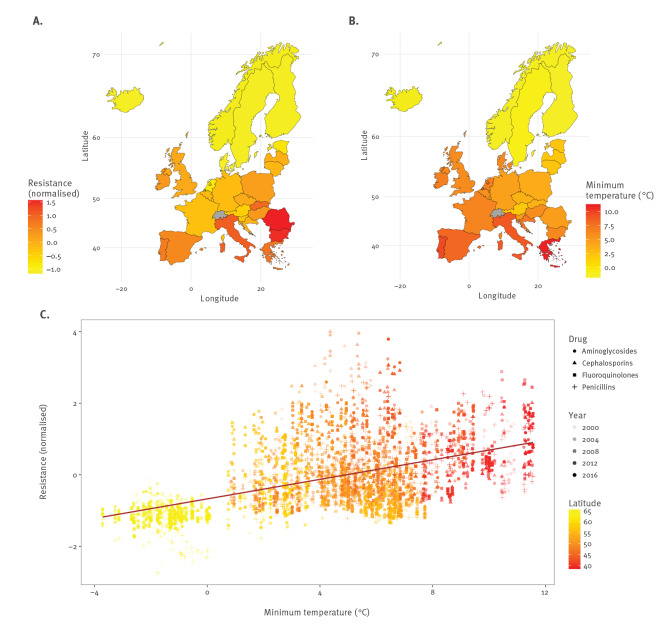
Antibiotic resistance increases with increasing minimum temperature, European Union/European Economic Area countries and the United Kingdom, 2000–2016

**Figure 2:**
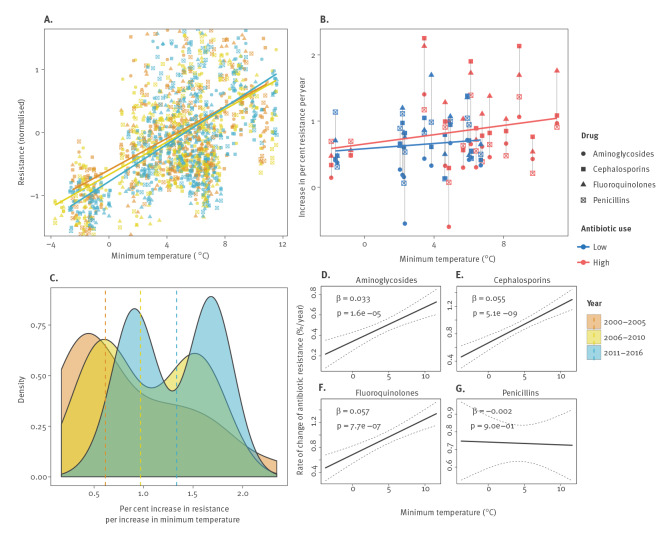
Change in the relationship between antibiotic resistance and minimum temperature over time for *Escherichia coli*, European Union/European Economic Area countries and the United Kingdom, 2000–2016

We found evidence of a positive linear association between minimum ambient temperature and antibiotic resistance across all countries, years, pathogens, and antibiotic subclasses (Figure 1), and observed that this positive linear relationship between temperature and resistance over geographies increased over time (Figure 2 A–C; Figures S7A–C; Figures S8A–C). In particular, note how the slope for the relationship between temperature and resistance increases with time (e.g. as years increase) in subfigures A (solid lines) and C (dashed lines), indicating that antibiotic resistance increases *faster* at higher temperatures. We also observed evidence for this relationship differential by antibiotic use, with a stronger relationship (larger slope) between temperature and increases in resistance at higher rates of higher antibiotic use (Figure 2B, Figure S7B, Figure S8B). Such evidence is consistent with previous findings across the US [14].

(A) Map with the mean normalised antibiotic resistance for three common pathogens (*Escherichia coli, Klebsiella pneumoniae,* and *Staphylococcus aureus*).

(B) Map with the annual average minimum temperature (°C) between 2000–2016, for 28 European countries.

(C) Unadjusted linear trend line estimating the linear relationship between annual average minimum temperature and normalised resistance, across all countries and pathogens and years. The transparency of the points in the scatter plot (represented by increasingly dark shades of grey) correspond to data collection year, and are coloured by latitude. Antibiotic subclasses are represented as geometric shapes.

(A) Normalised antibiotic resistance versus annual average minimum temperature (°C) for four antibiotic subclasses and all 28 countries, stratified by 5–6 year intervals.

(B) Speed of increase of antibiotic resistance versus annual average minimum temperature, stratified by average antibiotic consumption (low/high). Each country is represented by a collection of vertical points. The average antibiotic consumption for each country – over all drug classes and years – was compared to the median average antibiotic consumption across all 28 European countries. Countries with average antibiotic consumption less than the median across Europe were considered to have low antibiotic use (blue); with greater than the median, high antibiotic use (red).

(C) The changing association between annual average minimum temperature and antibiotic resistance over time. Distributions of the association between antibiotic resistance and minimum temperature (unadjusted linear model slopes) across multiple years and four antibiotic subclasses are shown, stratified by three time intervals (2000–2005; 2006–2010; 2011–2016). The median slope for each interval is marked by a vertical dashed line. Associations further to the right indicate stronger (steeper) relationships.

(D–G) Change of antibiotic resistance over time as a function of annual average minimum temperature for (D) aminoglycosides, (E) 3rd-generation cephalosporins, (F) fluoroquinolones, and (G) aminopenicillins, with 95% confidence intervals. Estimates for all antibiotics were obtained from multivariable models adjusting for country, minimum temperature (°C), year, population density (persons/km^2^), antibiotic consumption (defined daily doses /1,000 inhabitants/day), and the interaction between year and minimum temperature. Beta coefficients and p values are given for the interaction between minimum temperature and year, with 95% confidence intervals calculated using the standard error of Equation 3,

$\begin{array}{l} \sqrt{Var(b_{2})+Tmin^{2}\times Var(b_{3})+2Tmin\times Cov(b_{2},b_{3})} \end{array}$

where *b_2_* and *b_3_* are estimates of *β_2_* and *β_3_*. Equation 3 (from which the derived standard error is presented here) is described further in the main text of the current report.

### Effect of temperature on rates of change of antibiotic resistance

We developed a model relating temperature to antibiotic resistance accounting for time, antibiotic consumption, population density, and country-specific characteristics, described in the methods. By taking the time derivative of our primary model, Equation 1, we were able to assess how the rate of change of antibiotic resistance evolves in time for each pathogen and antibiotic subclass, as well as the extent to which cross-country variations in temperature and other predictors contributed to the rates of change of antibiotic resistance (Equation 2). Quantifying, for each year and country, the contribution of each predictor to the rate of change of antibiotic resistance, we found that differences in temporal rates of change of antibiotic resistance across geographies were driven mainly by spatial and temporal variations in minimum ambient temperature. In comparison contributions from other predictors that included temporal changes in population density, antibiotic consumption, and minimum temperature were smaller (Figure S9). For aminopenicillin resistance, the effect of minimum temperature was more modest but still larger than that of the other predictors (Figure S9). In Figure S10, we show that the variability in minimum temperature across countries in the data is much larger than the within-country temporal variability in minimum temperature over the 17-year time period, and countries experienced relatively small changes in population density and antibiotic consumption from year-to-year as shown in Figures S5, S6. Thus, for each pathogen and antibiotic subclass, our second result suggests that the most important contributor to differences in rates of change of antibiotic resistance across Europe is local ambient temperature.

In Figure S9, we also show that the magnitude of the term *Tmin* of Equation 2 is substantially larger than

$$\frac{d}{dt}(Tmin),\;\;\;\frac{d}{dt}(Population\;Density)$$

and

$$\frac{d}{dt}\log(Antibiotic\;\;Consumption)$$

and that the term *β_3_Tmin* is a good estimate of the term

$$\beta_{3}(Tmin+time*\frac{d}{dt}Tmin).$$

These findings suggest that Equation 2 can be approximated by an ‘effective’ equation (Equation 3) given by:

$$(3)\;\frac{d}{dt}Antibiotic\;\;resistance\;=\;\beta_{2}+\beta_{3}Tmin$$

Consequently, we found that warmer ambient minimum temperatures had a significant effect on the rates of increase (or in one instance decrease) of antibiotic resistance over time, a phenomenon consistent across most pathogens and antibiotic classes (Figures 2D–G, S7D–G, S8D–G). Over the study time period and controlling for national antibiotic consumption, population density, and constant country-specific confounders, we found that warmer countries, with 10°C larger values in average minimum temperature, experienced an increased rate of change of antibiotic resistance by 0.33%/year (p<0.001), 0.55%/year (p<0.001), and 0.57%/year (p<0.001) for *E. coli* resistance to aminoglycosides, 3rd-generation cephalosporins, and fluoroquinolones, respectively (Table, Figure 2D). We observed even faster rates of change of *K. pneumoniae* resistance to 3rd-generation cephalosporins and fluoroquinolones, of 0.9%/year (p<0.01) and 1.2%/year (p<0.01) for a 10°C increase in temperature across countries, respectively (Table, Figure S7D). Interestingly, we observed a decrease in the rate of change of antibiotic resistance across countries by approximately 0.4%/year (p<0.05), with a 10°C increase in minimum temperature, for meticillin resistant *S. aureus* (MRSA).

**Table:** Adjusted multivariable analyses to evaluate associations of antibiotic resistance with minimum temperature and other predictors, by pathogen and antibiotic subclass, European Union/European Economic Area countries and the United Kingdom, 2000–2016

Predictor	Coefficient (95% confidence interval)^a^			
	Aminoglycosides	Cephalosporins	Fluoroquinolones	Penicillins^b^
***Escherichia coli***				
Minimum temperature (°C)	−0.29 (−0.71 to 0.14)	−0.22 (−0.74 to 0.30)	−0.5 (−1.14 to 0.13)	−0.15 (−0.84 to 0.53)
Year	0.34^c^ (0.25 to 0.43)	0.67^c^ (0.56 to 0.77)	0.68^c^ (0.54 to 0.82)	0.74^c^ (0.59 to 0.89)
Minimum temperature (°C): year interaction	0.03^c^ (0.02 to 0.05)	0.05^c^ (0.04 to 0.07)	0.06^c^ (0.03 to 0.08)	0 (−0.03 to 0.02)
Antibiotic consumption (log DDD/1,000 persons)	0.32 (−0.00 to 0.64)	0.44^d^ (0.17 to 0.71)	2.02^c^ (1.03 to 3.00)	−0.62 (−2.07 to 0.84)
Population density (persons/km^2^)	−0.05 (−0.10 to 0.01)	−0.10^d^ (−0.17 to −0.03)	−0.06 (−0.14 to 0.02)	0.02 (−0.07 to 0.10)
R^2^	0.83	0.87	0.87	0.87
***Klebsiella pneumoniae***				
Minimum temperature (°C)	−1.07 (−2.29 to 0.15)	−1.23 (−2.52 to 0.06)	−1.34 (−2.93 to 0.24)	N/A
Year	0.56^d^ (0.21 to 0.91)	0.66^c^ (0.29 to 1.02)	0.92^c^ (0.48 to 1.36)	N/A
Minimum temperature (°C): year interaction	0.04 (−0.02 to 0.10)	0.09^d^ (0.03 to 0.15)	0.12^d^ (0.04 to 0.19)	N/A
Antibiotic consumption (log DDD/1,000 persons)	0.19 (−0.83 to 1.20)	0.90^d^ (0.25 to 1.54)	3.45^d^ (0.89 to 6.01)	N/A
Population density (persons/km^2^)	0.11 (−0.10 to 0.31)	0.04 (−0.17 to 0.26)	−0.28 (−0.55 to −0.02)	N/A
R^2^	0.93	0.94	0.87	N/A
***Staphylococcus aureus***				
Minimum temperature (°C)	N/A	N/A	N/A	0.7 (−0.25 to 1.65)
Year	N/A	N/A	N/A	−0.04 (−0.23 to 0.15)
Minimum temperature (°C): year interaction	N/A	N/A	N/A	−0.04^e^ (−0.07 to 0.00)
Antibiotic consumption (log DDD/1,000 persons)	N/A	N/A	N/A	0.34 (−1.70 to 2.38)
Population density (persons/km^2^)	N/A	N/A	N/A	−0.29^c^ (−0.41 to −0.17)
R^2^	N/A	N/A	N/A	0.87

N/A: data not available; DDD: number of defined daily doses (DDD) per day, from combined inpatient and outpatient sources.

^a^ Except for the rows showing the R^2^.

^b^ Penicillin resistance in *E.coli* was measured as resistance to aminopenicillins, and for *S. aureu*s as resistance to meticillin.

^c^ p < 0.001.

^d^ p < 0.01.

^e^ p < 0.05.

Coefficients with standard errors (95% confidence intervals) are adjusted for country, minimum temperature, year, population density, antibiotic consumption, and the interaction between year and minimum temperature. For interpretability, year is zeroed at baseline (2000). A natural log transform was applied to antibiotic consumption to improve linear fit. All available pathogen–antibiotic combinations of three pathogens (*E. coli*, *K. pneumoniae*, and *S. aureus*) and four antibiotic subclasses (aminoglycosides, 3rd-generation cephalosporins, fluoroquinolones, and penicillins) were analysed.

We also observed that higher values of antibiotic consumption, thought to have important roles in conferring antibiotic resistance, including through population-level selective pressure [14,22], were generally associated with higher values of antibiotic resistance to cephalosporins and fluoroquinolones for the pathogens *E. coli* and *K. pneumoniae* (Table; Figure S11). For a few pathogen and antibiotic combinations, lower resistance was associated with more densely populated countries (p<0.01, Table). Across pathogens, the relationship between antibiotic resistance and minimum temperature did not substantively change by median antibiotic consumption or by tertile of population density (Figure S12).

### Long-term effect of temperature

Our results were almost identical when we conducted our statistical analysis on two different temperature data sources and two distinct spatial resolutions (country- and city-level), confirming that our findings are robust (Table, Supplementary tables, S2, S3). Moreover, by removing the within-country temporal variability in minimum temperature, and conducting our analysis using a fixed 17-year average minimum temperature we recovered our original findings (Table S4). The latter result establishes the presence of a long-term effect of minimum temperature on the rate of increase of antibiotic resistance. Our attempts to identify potential short-term climatic influences of our set of predictors on antibiotic resistance, via time-series analysis, did not lead to consistent patterns. For this, we conducted a post-hoc analysis on the direction of the changes (time derivatives), from one year to the next, of minimum temperature, antibiotic consumption, and antibiotic resistance. We calculated the proportion of times for which the direction (increase/decrease) of annual changes of antibiotic resistance and the predictors were the same. This metric, often called ‘hit rate’, captured the congruence in the directional movement of trends in antibiotic resistance, minimum temperature, and antibiotic consumption. Based on these short-term trends analyses, there was no general pattern suggesting that short-term temporal deviations in temperature and antibiotic consumption, either synchronously or lagged (by 1, 2 or 3 years), could explain short-term temporal deviations in antibiotic resistance, on average across countries (mean hit rates < 60%; Figure S13). Note that a hit rate of about 50% suggests that half of the times one of the signals increased, the other one decreased. So a strong signal takes place when the majority of the times both move up or down. Within some countries and for some pathogens and antibiotic subclasses, there may be strong signatures of short-term trends (Figures S14–S16), which future analyses may explore.

## Discussion

Our ecologic study presents evidence that the rate at which antibiotic resistance accumulates over time is associated with ambient temperature across Europe among a collection of three pathogens and four antibiotic subclasses. While our findings should not be generalised to other bacterial pathogens and antibiotic classes without further analysis, they may help understand the currently observed geographic distribution of antibiotic resistance prevalence in Europe, which shows warmer areas experiencing higher levels of antibiotic resistance. We found that warmer ambient temperatures are generally associated with larger rates of increase of resistance for *E. coli* and *K. pneumoniae*, and for larger rates of decrease for *S. aureus.*

These findings for *S. aureus,* which appear contradictory to those of *E. coli* and *K. pneumoniae*, may reflect concerted efforts to reduce MRSA infections in countries with high endemic MRSA prevalence. These efforts to curb MRSA have been well documented in Europe [23] where many countries have instituted successful programmes that target healthcare-associated MRSA (HA-MRSA) transmission and infections through enhanced infection prevention and control practices. These efforts may be unique to MRSA and thus are unlikely to impact other pathogens and susceptibilities.

Interestingly, we found a negative association between antibiotic resistance and population density, which is contrary to previous findings in the US, where antibiotic resistance was associated with higher population density [14]. Further studies should investigate the consistency of relationships between population density and resistance across geographies, though one possible explanation to these findings in Europe could be the relationship between farming and antibiotic use in less densely populated areas, which may contribute to the observed negative association.

The role of climate and infectious diseases has been a major topic of research in the last decade. However, much of this work has focused on the impact of vector-borne diseases (e.g. malaria and dengue), as well as diseases related to water and sanitation (e.g. cholera) [13]. To date, there has been a paucity of literature on the relationship between climate factors and population-level antibiotic resistance. Two exceptions are an ecologic study across the US [14], which due to limited temporal resolution could not assess trends over time, and a recent ecologic study across Europe [24], which found evidence for a relationship between temperature and the geographic distribution of antibiotic resistance, but also did not assess trends in time. Our study differs fundamentally from the latter study in that it leverages longitudinal data to offer deeper insights into rates of change of resistance across Europe – how fast resistance is spreading as a function of time and temperature – rather than purely geographic distributions as have been previously described. Aside from these two studies, a few other investigations have touched on the potential importance of temperature in driving antibiotic resistance. One recent country-level analysis in Europe did not explicitly assess the effects of temperature on temporal trends in antibiotic resistance, but did find that increasing distance from the equator was a predictor of lower risk of resistance of one pathogen genus, *Acinetobacter* spp, to one antibiotic class, carbapenems [25]. Further, a study using data from 103 countries found that temperature was positively correlated (univariate) with antibiotic resistance indices, but did not isolate the individual effect of temperature on resistance in multivariable models, instead combining temperature and precipitation data to create a single ‘climate’ variable [26]. Further, this study did not assess trends in time.

Compared to previous studies, our study offers evidence for the positive relationship between temperature and antibiotic resistance over a large geographic and temporal scale, across three bacterial pathogens and four drug classes and using data that includes the most comprehensive antibiotic resistance and consumption data from national surveillance networks, the gold standard for this kind of data. Other datasets offer the future promise of evaluating the potential impact of climate on antibiotic resistance on a global scale [5,27].

While we have a limited understanding of the transmission networks that give rise to the spread of antibiotic resistant organisms and mechanisms of resistance, there is a general understanding that this is a complex process, connecting agriculture, animals, humans, and the broader environment [28]. Temperature plays an important role in many of these settings, and there are a number of potential mechanisms that could support a biological effect of temperature on antibiotic resistance distribution and transmission. Antibiotic resistant bacteria have either intrinsic or acquired mechanisms that render them non-susceptible to particular types of antibiotics, and these bacteria can exist in both the environment and hosts (e.g. carriage within flora of humans and animals) [29]. On a bacterial level, mechanisms of resistance can either be shared vertically (through strain replication) or horizontally (e.g. from other bacteria, viruses, or the environment) [29,30]. Temperature thus has the potential to act on any number of these resistance acquisition and transfer mechanisms, both within and between hosts and environments. For example, increased carriage in humans and animals could support increased transmission of resistant strains of bacteria, and carriage of antibiotic resistant organisms has been shown to be influenced by season/temperature [11]. Moreover, horizontal gene transfer (including the transfer of antibiotic resistance genes between bacteria) is known to be temperature dependent, and previous work has shown that optimal temperatures for transfer of the carbapenemase New Delhi metallo-β-lactamase 1 (NDM-1) are similar to local ambient temperatures [10]. Increased bacterial growth rates at higher temperatures could facilitate overall transmission and selection by antibiotic consumption [9]. The environment is thought to be a major reservoir of antibiotic resistance [31], and it is also possible that the environmental resistome (the collection of all resistance genes) could become more diverse in a warming climate. Lastly, temperature may have an impact on other important human socio-behavioral effects (e.g. gatherings) which could also alter the transmission of antibiotic resistant organisms. While much of the resistance data obtained from surveillance networks are likely from hospital (nosocomial) sources, we would expect much of the flora leading to infection to have been derived from community colonisation [32,33], upholding the hypotheses raised here.

Limitations to our study include issues inherent to data collection and databases used, such as gaps in national data reporting over time, both systematic (e.g. Lithuania and Latvia not reporting resistance data until 2005−2006) and random (e.g. Portugal missing resistance data in 2007). In this study, gaps in data reporting and collection apply in particular to resistance data sourced from EARS-Net and antibiotic consumption data sourced from ESAC-Net. To address the systematically missing data for countries from 2000–2005, we report a separate analysis for 2006–2016 and show that our original findings hold (Table S5). In addition to gaps in data collection, methods and measures to evaluate antibiotic consumption and antibiotic resistance may change over time (see Supporting Information, ‘Additional Limitations’). Changes in breakpoints are infrequent occurrences for any given class and do not often occur in synchrony with all other classes, which makes them difficult to adjust for in an analysis. However, their impact on our results is unlikely for the following reasons: we do not expect breakpoints to change as a function of temperature (our exposure); our findings are robust to multiple classes of antibiotics; and breakpoint changes based on the introduction of a new drug class are not necessarily reflected in antibiotic consumption immediately after introduction, and this likely varies by country.

Our study dataset was also limited to annual national antibiotic resistance levels, restricting our geographic analysis to the country level and our temporal analysis to the yearly level. As such, evaluating associations on smaller geographic (e.g. state/city) or time (e.g. seasonal) scales was not possible. Evaluating intra-year temperature fluctuations and resistance patterns may lead to additional important insights, and should be pursued with the availability of more granular data. Lastly, the ability to quantify and include potential confounders related to policy (such as historical efforts to curb MRSA) is currently limited, but would improve the ability to test and control for the effects of policy implementation on the rates of antibiotic resistance spread over the years. As a sensitivity analysis (Table S6) we introduced two time-varying predictors (i) national gross domestic product (GDP) and (ii) total national health expenditure, and this resulted in no notable changes in the study conclusions.

If future studies confirm that the rate at which antibiotic resistance accumulates in human pathogens is influenced by temperature at the global scale, directly or indirectly, then our findings suggest that future increases in global temperature may yield faster increases in antibiotic resistance [34]. This finding was raised speculatively in a report examining the geographic distribution of antibiotic resistance in the US [14], and an immediate next step could involve amassing more years of data on resistance in the US to confirm the findings of the present study.

Estimating the health and economic burden of antibiotic resistance globally is challenging: one extreme estimate suggests that attributable mortality due to antibiotic resistance could be on the order of ‘millions’ annually by 2050, with annual economic burden in excess of ‘billions’ of US dollars [35-38]. While we cannot infer causality from our study, we have demonstrated that temperature may play an important role in modulating the rate of change of antibiotic resistance in an area, and this may explain the geographic differences that have been observed in cross-sectional studies. While our approach of using country-wide minimum temperatures was done to provide comparability with national antibiotic resistance data and could lead to blunted associations, these would still likely only lead to underestimates. Further, our sensitivity analyses using different data sources and spatial resolutions for minimum temperature confirm the consistency of our findings, strengthening their likelihood and providing evidence for a long-term effect of temperature on antibiotic resistance. Regardless of the methodology used to estimate the prevalence of antibiotic resistance globally, failure to account for potentially relevant factors such as temperature could lead to underestimates. We hope this work will drive further avenues of research to investigate the role of climate as well as other sociodemographic factors on the distribution and spread of antibiotic resistance.

## Supplementary Data

4193000 Supplementary Material
