# Author’s correction for Euro Surveill. 2020;25(45)

**DOI:** 10.2807/1560-7917.ES.2020.25.46.201119a

**Published:** 2020-11-19

**Authors:** Eurosurveillance editorial team

**Affiliations:** 1 European Centre for Disease Prevention and Control (ECDC), Stockholm, Sweden

In the article ‘Rates of increase of antibiotic resistance and ambient temperature in Europe: a cross-national analysis of 28 countries between 2000 and 2016’ by McGough et al. published on 12 November 2020, at the request of the authors, *Klebsiella pneumoniae* (Gram-positive) was corrected to *Klebsiella pneumoniae* (Gram-negative) and *Staphylococcus aureus* (Gram-negative) was corrected to *Staphylococcus aureus* (Gram-positive). The changes were made on 19 November 2020.

