# Supplementary material for: Rates of increase of antibiotic resistance and ambient temperature in Europe: a cross-national analysis of 28 countries between 2000 and 2016
Source: Euro Surveill. 2020 Nov 12;25(45):1900414. doi: 10.2807/1560-7917.ES.2020.25.45.1900414 (PMC7667635; doi:10.2807/1560-7917.ES.2020.25.45.1900414)
Supplement: Supplementary Material [file 1900414_Supplement.pdf]

This supplementary material is hosted by *Eurosurveillance* as supporting information alongside the article “Rates of increase of antibiotic resistance and ambient temperature in Europe: a cross-national analysis of 28 countries between 2000 and 2016,” on behalf of the authors, who remain responsible for the accuracy and appropriateness of the content. The same standards for ethics, copyright, attributions and permissions as for the article apply. Supplements are not edited by *Eurosurveillance* and the journal is not responsible for the maintenance of any links or email addresses provided therein.

Supplementary Materials

Additional Limitations

In this paper, we performed an ecologic analysis of association between country level outcomes with country level predictors, and as such we cannot infer causality. When choosing measures of antibiotic consumption, we selected only those corresponding to the class of antibiotic susceptibility (or resistance), i.e. we related fluoroquinolone consumption to fluoroquinolone resistance. We did seek to evaluate non-linear forms of consumption (in the forms of splines), however we did not seek to evaluate the impacts of *other* classes of antibiotics (to model co-selection) due to potential complexity and uncertainty of effect. This includes use of antimicrobials for food animals.

The antibiotic resistance and antibiotic consumption data have a number of limitations which are acknowledged by their source. Namely, the degree of data comprehensiveness across countries is initially sparse, but generally increases over time. The methods/measures to evaluate antibiotic consumption and antibiotic resistance may vary by country and may change over time. Most relevant to this paper would be changes in breakpoints over time. However, consistency of findings supports likely limited impacts of these changes, and the data are sourced from the most comprehensive database available. Lastly, the bacterial isolate sources (for determining antibiotic prevalence) come from usually sterile sites and are thus may be more reflective of severe and/or hospital origin infections.

Sensitivity Analyses

In order to explore potential non-linear relationships between antimicrobial consumption and antibiotic resistance, we also developed multivariable models with the relationship between antibiotic resistance and antibiotic consumption modeled as a natural cubic spline with k=3 knots, in order to account for potentially non-linear relationships. This sensitivity analysis did not demonstrate material differences in our findings (Table S2).

In addition, in order to identify long-term climatic influences on antibiotic resistance, we repeated the original analysis using a fixed value for temperature, representing the average minimum temperature over the 17-year study period. We recovered nearly identical coefficients and p-values compared to the original analysis (Table S5).

Data Use Disclaimer

The views and opinions of the authors expressed herein do not necessarily state or reflect those of ECDC. The accuracy of the authors' statistical analysis and the findings they report are not the responsibility of ECDC. ECDC is not responsible for conclusions or opinions drawn from the data provided. ECDC is not responsible for the correctness of the data and for data management, data merging and data collation after provision of the data. ECDC shall not be held liable for improper or incorrect use of the data.

Table S1. List of cities or sources from which weather station data were obtained. Weather stations were available for 26 of the 28 countries in the main analysis, and data were compiled by the European Climate Data & Assessment (ECD&A) project of the Royal Netherlands Meteorological Institute (KNMI).

Country	Weather Station City/Source
Austria	Vienna
Belgium	Uccle
Croatia	Zagreb
Denmark	Copenhagen
Estonia	Voru
Finland	Helsinki
France	Paris
Germany	Berlin
Greece	Heraklion
Hungary	Budapest
Iceland	Reykjavik
Ireland	Valentia Observatory
Italy	Rome
Latvia	Liepja
Lithuania	Kaunas
Luxembourg	Luxembourg Airport
Netherlands	Maastricht
Norway	Oslo
Poland	Warsaw
Portugal	Lisbon
Romania	Bucharest
Slovakia	Hurbanovo
Slovenia	Ljubljana
Spain	Madrid
Sweden	Stockholm
United Kingdom	Central England Temperature (CET)

Table S2. Adjusted multivariable analyses by pathogen and antibiotic class, for European capital cities using MERRA-2 reanalysis weather data. Coefficients with standard errors (95% confidence intervals) were adjusted for country, minimum temperature, year, population density, antibiotic consumption, and the interaction between year and minimum temperature. For interpretability, year was zeroed at baseline (2000). A log transform was applied to antibiotic consumption to improve linear fit. All available pathogen-antibiotic combinations of 3 pathogens (*E. coli*, *K. pneumoniae*, and *S. aureus*) and 4 antibiotic classes (aminoglycosides, 3rd-generation cephalosporins, fluoroquinolones, and penicillins) were analyzed. For each capital city, daily minimum temperature from MERRA-2 were obtained from the 0.5° x 0.625° grid cell covering the centroid of the city, and the annual mean for each country was computed for each calendar year. Penicillin resistance in *E.coli* was measured as resistance to aminopenicillins, and for *S. aureu*s as methicillin resistance.

	Aminoglycosides	Cephalosporins	Fluoroquinolones	Penicillins
E. coli				
Minimum temperature (°C)	-0.33	-0.26	-0.31	-0.05
	(-0.75, 0.08)	(-0.77, 0.26)	(-0.94, 0.32)	(-0.71, 0.62)
Year	0.26 ***	0.67 ***	0.72 ***	0.82 ***
	(0.15, 0.37)	(0.53, 0.80)	(0.55, 0.90)	(0.64, 1.00)
Minimum temperature (°C): Year Interaction	0.04 ***	0.04 ***	0.04 **	-0.01
	(0.02, 0.06)	(0.02, 0.06)	(0.01, 0.06)	(-0.04, 0.01)
Antibiotic consumption (log DDD/1000 persons)	0.26	0.49 ***	2.00 ***	-0.57
	(-0.07, 0.58)	(0.22, 0.77)	(0.99, 3.01)	(-2.03, 0.89)
Population density (log persons/km^2)	-0.04	-0.08 *	-0.04	0.02
	(-0.09, 0.02)	(-0.15, -0.02)	(-0.12, 0.05)	(-0.07, 0.11)
R^2	0.83	0.86	0.86	0.87
K. pneumoniae				
Minimum temperature (°C)	-0.87	-1	-0.86	
	(-2.09, 0.35)	(-2.30, 0.30)	(-2.47, 0.74)	
Year	0.67 **	0.67 **	1.10 ***	
	(0.24, 1.09)	(0.21, 1.13)	(0.54, 1.66)	
Minimum temperature (°C): Year Interaction	0.01	0.07 *	0.06	
	(-0.05, 0.08)	(0.00, 0.14)	(-0.02, 0.15)	
Antibiotic consumption (log DDD/1000 persons)	0.24	0.95 **	3.56 **	
	(-0.77, 1.25)	(0.30, 1.60)	(0.96, 6.16)	
Population density (log persons/km^2)	0.12	0.07	-0.24	
	(-0.09, 0.33)	(-0.15, 0.28)	(-0.51, 0.02)	
R^2	0.93	0.94	0.87	
S. aureus				
Minimum temperature (°C)				-0.01
				(-0.94, 0.92)
Year				-0.31 *
				(-0.55, -0.07)
Minimum temperature (°C): Year Interaction				0.02
				(-0.01, 0.06)
Antibiotic consumption (log DDD/1000 persons)				0.08
				(-1.97, 2.13)
Population density (log persons/km^2)				-0.33 ***
				(-0.45, -0.21)
R^2				0.87

*** $p < 0.001$; ** $p < 0.01$; * $p < 0.05$.

Table S3. Adjusted multivariable analyses by pathogen and antibiotic class, for European cities using weather station data. Coefficients with standard errors (95% confidence intervals) were adjusted for country, minimum temperature, year, population density, antibiotic consumption, and the interaction between year and minimum temperature. For interpretability, year was zeroed at baseline (2000). A log transform was applied to antibiotic consumption to improve linear fit. All available pathogen-antibiotic combinations of 3 pathogens (*E. coli*, *K. pneumoniae*, and *S. aureus*) and 4 antibiotic classes (aminoglycosides, 3rd-generation cephalosporins, fluoroquinolones, and penicillins) were analyzed. Penicillin resistance in *E.coli* was measured as resistance to aminopenicillins, and for *S. aureus* as methicillin resistance. Weather station data for capital or populous cities were obtained from the European Climate Data & Assessment (ECD&A) project of the Royal Netherlands Meteorological Institute (KNMI). 26 of the 28 countries had available weather station data, and the list of cities used for each country can be found in Table S1.

	**Aminoglycosides**	**Cephalosporins**	**Fluoroquinolones**	**Penicillins**
E. coli				
Minimum temperature (°C)	-0.48 *	-0.34	-0.66	-0.6
	(-0.89, -0.07)	(-0.85, 0.17)	(-1.34, 0.01)	(-1.31, 0.11)
Year	0.14 *	0.46 ***	0.53 ***	0.64 ***
	(0.02, 0.26)	(0.31, 0.61)	(0.32, 0.73)	(0.42, 0.85)
Minimum temperature (°C): Year Interaction	0.05 ***	0.05 ***	0.06 ***	0.01
	(0.03, 0.06)	(0.03, 0.07)	(0.03, 0.08)	(-0.01, 0.04)
Antibiotic consumption (log DDD/1000 persons)	0.49 **	0.36 **	1.58 **	-0.94
	(0.14, 0.84)	(0.10, 0.61)	(0.54, 2.63)	(-2.37, 0.49)
Population density (log persons/km²)	-0.03	-0.03	-0.01	0.04
	(-0.08, 0.02)	(-0.09, 0.03)	(-0.09, 0.07)	(-0.05, 0.12)
R^2	0.81	0.84	0.87	0.88
K. pneumoniae				
Minimum temperature (°C)	-1.2	-1.39	-0.5	
	(-2.59, 0.20)	(-2.86, 0.08)	(-2.33, 1.33)	
Year	0.51	0.47	1.17 **	
	(-0.04, 1.06)	(-0.11, 1.05)	(0.46, 1.89)	
Minimum temperature (°C): Year Interaction	0.03	0.07 *	0.04	
	(-0.04, 0.10)	(0.00, 0.15)	(-0.05, 0.13)	
Antibiotic consumption (log DDD/1000 persons)	-0.46	0.82 *	3.64 **	
	(-1.70, 0.78)	(0.13, 1.50)	(0.97, 6.31)	
Population density (log persons/km²)	0.13	0.1	-0.25	
	(-0.08, 0.35)	(-0.12, 0.32)	(-0.53, 0.03)	
R^2	0.92	0.93	0.86	
S. aureus				
Minimum temperature (°C)				-0.32
				(-1.33, 0.70)
Year				-0.04
				(-0.34, 0.25)
Minimum temperature (°C): Year Interaction				-0.01
				(-0.04, 0.03)
Antibiotic consumption (log DDD/1000 persons)				0.75
				(-1.33, 2.84)
Population density (log persons/km²)				-0.40 ***
				(-0.52, -0.28)
R^2				0.88

*** $p < 0.001$; ** $p < 0.01$; * $p < 0.05$.

Table S4. Adjusted multivariable analyses by pathogen and antibiotic class, with a cubic spline for antibiotic consumption. Coefficients with standard errors (95% confidence intervals) are adjusted for country, minimum temperature, year, population density, antibiotic consumption, and the interaction between year and minimum temperature. For interpretability, year is zeroed at baseline (2000). To improve linear fit, antibiotic consumption was modeled with a natural cubic spline (k=3 knots). All available pathogen-antibiotic combinations of 3 pathogens (*E. coli*, *K. pneumoniae*, and *S. aureus*) and 4 antibiotic classes (aminoglycosides, 3rd-generation cephalosporins, fluoroquinolones, and penicillins) were analyzed. Penicillin resistance in *E.coli* was measured as resistance to aminopenicillins, and for *S. aureus* as methicillin resistance.

	Aminoglycosides	Cephalosporins	Fluoroquinolones	Penicillins
E. coli				
Minimum temperature (°C)	-0.28	-0.20	-0.45	-0.02
	(-0.70, 0.14)	(-0.68, 0.29)	(-1.08, 0.18)	(-0.70, 0.66)
Year	0.35 ***	0.63 ***	0.74 ***	0.71 ***
	(0.26, 0.44)	(0.53, 0.73)	(0.59, 0.89)	(0.56, 0.87)
Population density (persons/km²)	-0.04	-0.06	-0.08	0.03
	(-0.09, 0.02)	(-0.12, 0.00)	(-0.16, 0.01)	(-0.06, 0.12)
Minimum temperature (°C): Year Interaction	0.03 ***	0.05 ***	0.05 ***	-0.01
	(0.02, 0.05)	(0.03, 0.07)	(0.02, 0.07)	(-0.03, 0.01)
ns(Antibiotic consumption)1 (DDD/1000 persons)	0.09	0.82	0.71	-6.26*
	(-1.32, 1.51)	(-1.55, 3.19)	(-3.18, 4.59)	(-12.5, -0.01)
ns(Antibiotic consumption)2 (DDD/1000 persons)	5.50**	37.64***	11.38	-2.20
	(1.54, 9.46)	(28.1, 47.18)	(6.59, 16.17)	(-9.02, 4.62)
ns(Antibiotic consumption)3 (DDD/1000 persons)	0.09	24.42***	5.38	-19.99**
	(-3.66, 3.85)	(16.65, 32.18)	(-3.57, 14.34)	(-32.99, -6.99)
ns(Antibiotic consumption)4 (DDD/1000 persons)	-1.35	10.70**	0.03	-1.72
	(-5.32, 2.62)	(4.17, 17.23)	(-7.32, 7.37)	(-10.03, 6.6)
R 2	0.83	0.89	0.87	0.88
K. pneumoniae				
Minimum temperature (°C)	-1.07	-1.36 *	-0.96	
	(-2.30, 0.17)	(-2.66, -0.07)	(-2.54, 0.62)	
Year	0.60 ***	0.63 **	0.98 ***	
	(0.25, 0.95)	(0.25, 1.00)	(0.53, 1.43)	
Population density (persons/km²)	-1.99	1.83	2.78	
	(-5.50, 1.51)	(-4.16, 7.82)	(-8.00, 13.56)	
Minimum temperature (°C): Year Interaction	0.04	0.10 **	0.09 *	
	(-0.02, 0.10)	(0.03, 0.16)	(0.02, 0.17)	
ns(Antibiotic consumption)1 (DDD/1000 persons)	2.63	7.83*	4.74	
	(-1.96, 7.22)	(1.29, 14.37)	(-6.39, 15.87)	
ns(Antibiotic consumption)2 (DDD/1000 persons)	-2.13	24.17	17.36*	
	(-13.15, 8.9)	(-1.03, 49.38)	(3.99, 30.72)	
ns(Antibiotic consumption)3 (DDD/1000 persons)	-1.53	21.27*	9.50	
	(-13.22, 10.15)	(0.94, 41.61)	(-15.94, 34.95)	
ns(Antibiotic consumption)4 (DDD/1000 persons)	-2.16	9.07	-0.78	
	(-12.75, 8.42)	(-6.99, 25.13)	(-17.23, 15.68)	
R 2	0.93	0.94	0.87	
S. aureus				
Minimum temperature (°C)				0.71
				(-0.25, 1.66)
Year				-0.11
				(-0.30, 0.08)
Population density (persons/km²)				-9.75 **
				(-16.66, -2.83)
Minimum temperature (°C): Year Interaction				-0.03
				(-0.06, 0.00)
ns(Antibiotic consumption)1 (DDD/1000 persons)				9.77*
				(0.93, 18.61)
ns(Antibiotic consumption)2 (DDD/1000 persons)				1.97
				(-7.77, 11.71)
ns(Antibiotic consumption)3 (DDD/1000 persons)				17.22
				(-1.17, 35.61)
ns(Antibiotic consumption)4 (DDD/1000 persons)				0.35
				(-11.58, 12.28)
R 2				0.88

*** $p < 0.001$; ** $p < 0.01$; * $p < 0.05$.

Table S5. Adjusted multivariable analyses by pathogen and antibiotic class, using a fixed 17-year average for minimum temperature. Coefficients with standard errors (95% confidence intervals) are adjusted for country, the 17-year average minimum temperature, year, population density, antibiotic consumption, and the interaction between year and the 17-year average minimum temperature. For interpretability, year is zeroed at baseline (2000). A log transform was applied to antibiotic consumption to improve linear fit. All available pathogen-antibiotic combinations of 3 pathogens (*E. coli*, *K. pneumoniae*, and *S. aureus*) and 4 antibiotic classes (aminoglycosides, 3rd-generation cephalosporins, fluoroquinolones, and penicillins) were analyzed. Penicillin resistance in *E.coli* was measured as resistance to aminopenicillins, and for *S. aureus* as methicillin resistance.

	Aminoglycosides	Cephalosporins	Fluoroquinolones	Penicillins
E. coli				
Minimum temperature (°C)	-0.29	-0.22	-0.5	-0.15
	(-0.71, 0.14)	(-0.74, 0.30)	(-1.14, 0.13)	(-0.84, 0.53)
Year	0.34***	0.67***	0.68***	0.74***
	(0.25, 0.43)	(0.56, 0.77)	(0.54, 0.82)	(0.59, 0.89)
Minimum temperature (°C): Year Interaction	0.03***	0.05***	0.06***	-0.002
	(0.02, 0.05)	(0.04, 0.07)	(0.03, 0.08)	(-0.03, 0.02)
Antibiotic consumption (log DDD/1000 persons)	0.32*	0.44***	2.02***	-0.62
	(-0.005, 0.64)	(0.17, 0.71)	(1.03, 3.00)	(-2.07, 0.84)
Population density (persons/km^2)	-0.05	-0.10***	-0.06	0.02
	(-0.10, 0.01)	(-0.17, -0.03)	(-0.14, 0.02)	(-0.07, 0.10)
R^2	0.83	0.87	0.87	0.87
K. pneumoniae				
Minimum temperature (°C)	-1.07*	-1.23*	-1.34*	
	(-2.29, 0.15)	(-2.52, 0.06)	(-2.93, 0.24)	
Year	0.56***	0.66***	0.92***	
	(0.21, 0.91)	(0.29, 1.02)	(0.48, 1.36)	
Minimum temperature (°C): Year Interaction	0.04	0.09***	0.12***	
	(-0.02, 0.10)	(0.03, 0.15)	(0.04, 0.19)	
Antibiotic consumption (log DDD/1000 persons)	0.19	0.90***	3.45***	
	(-0.83, 1.20)	(0.25, 1.54)	(0.89, 6.01)	
Population density (persons/km^2)	0.11	0.04	-0.28**	
	(-0.10, 0.31)	(-0.17, 0.26)	(-0.55, -0.02)	
R^2	0.93	0.94	0.87	
S. aureus				
Minimum temperature (°C)				0.7
				(-0.25, 1.65)
Year				-0.04
				(-0.23, 0.15)
Minimum temperature (°C): Year Interaction				-0.04**
				(-0.07, -0.004)
Antibiotic consumption (log DDD/1000 persons)				0.34
				(-1.70, 2.38)
Population density (persons/km^2)				-0.29***
				(-0.41, -0.17)
R^2				0.87

*** $p < 0.001$; ** $p < 0.01$; * $p < 0.05$.

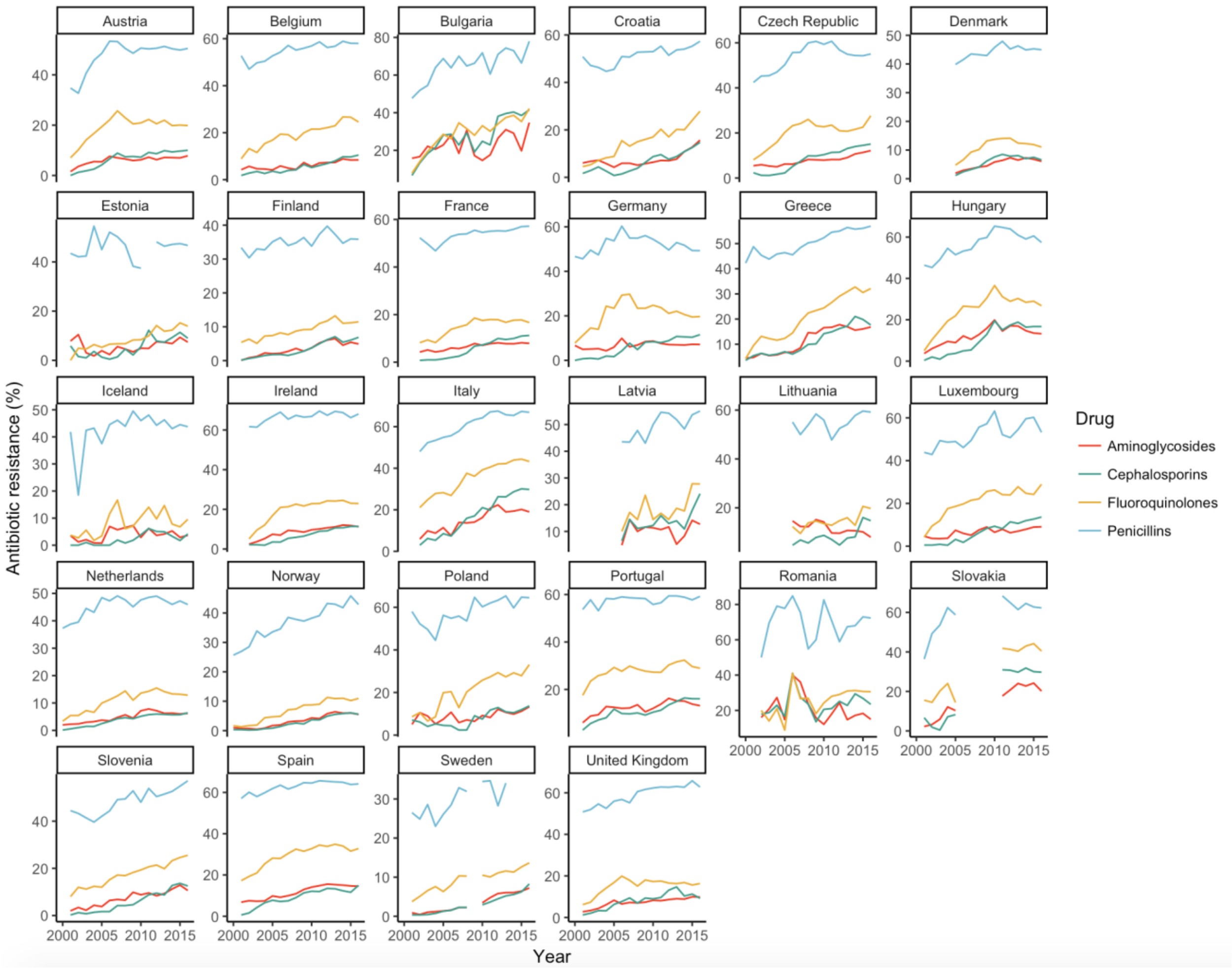

Figure S1. Trends in antibiotic resistance (%) for *E. coli* between 2000-2016 across 28 countries in Europe. Antibiotic classes are represented by colored lines.

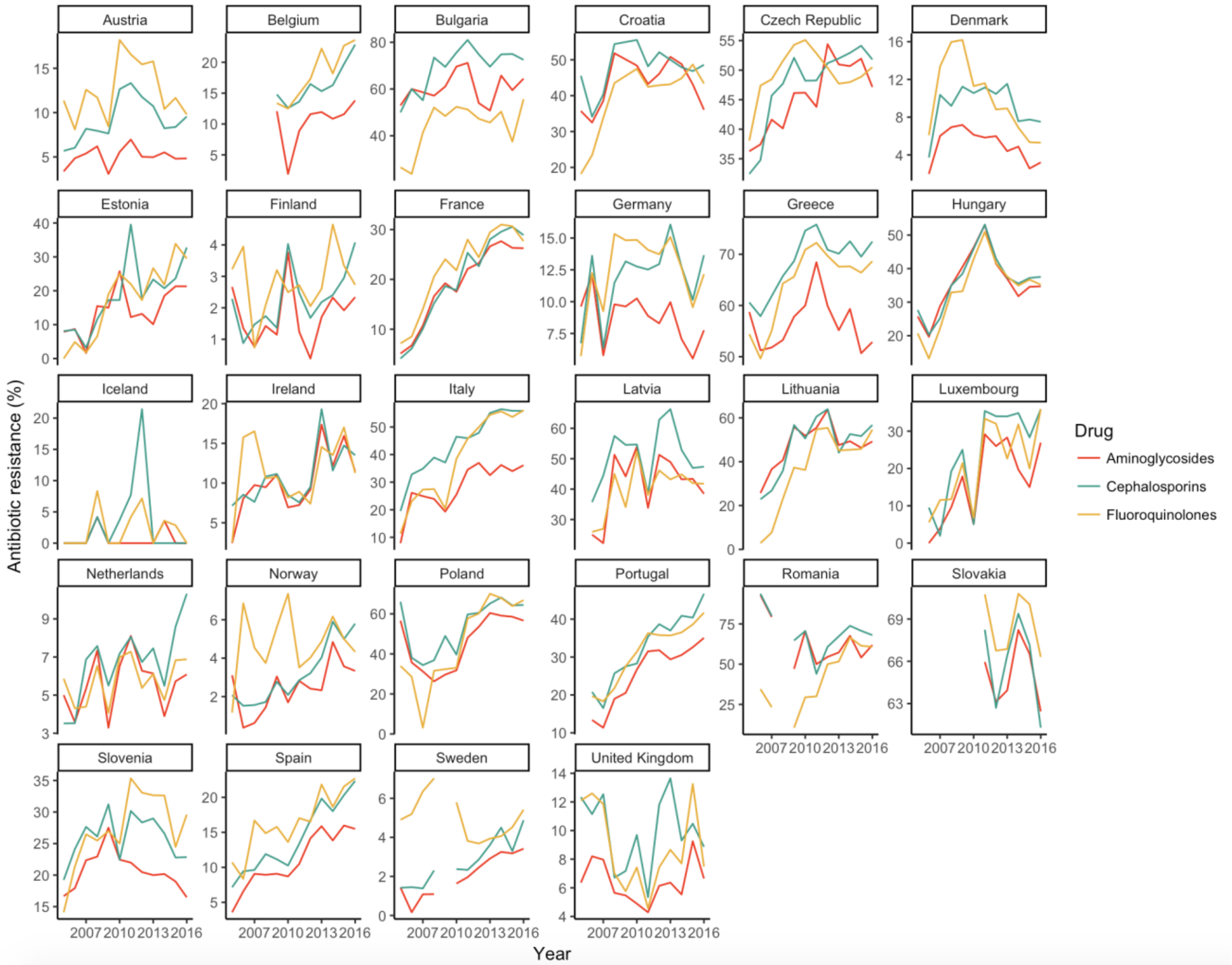

Figure S2. Trends in antibiotic resistance (%) for *K. pneumoniae* between 2000-2016 across 28 countries in Europe. Antibiotic classes are represented by colored lines.

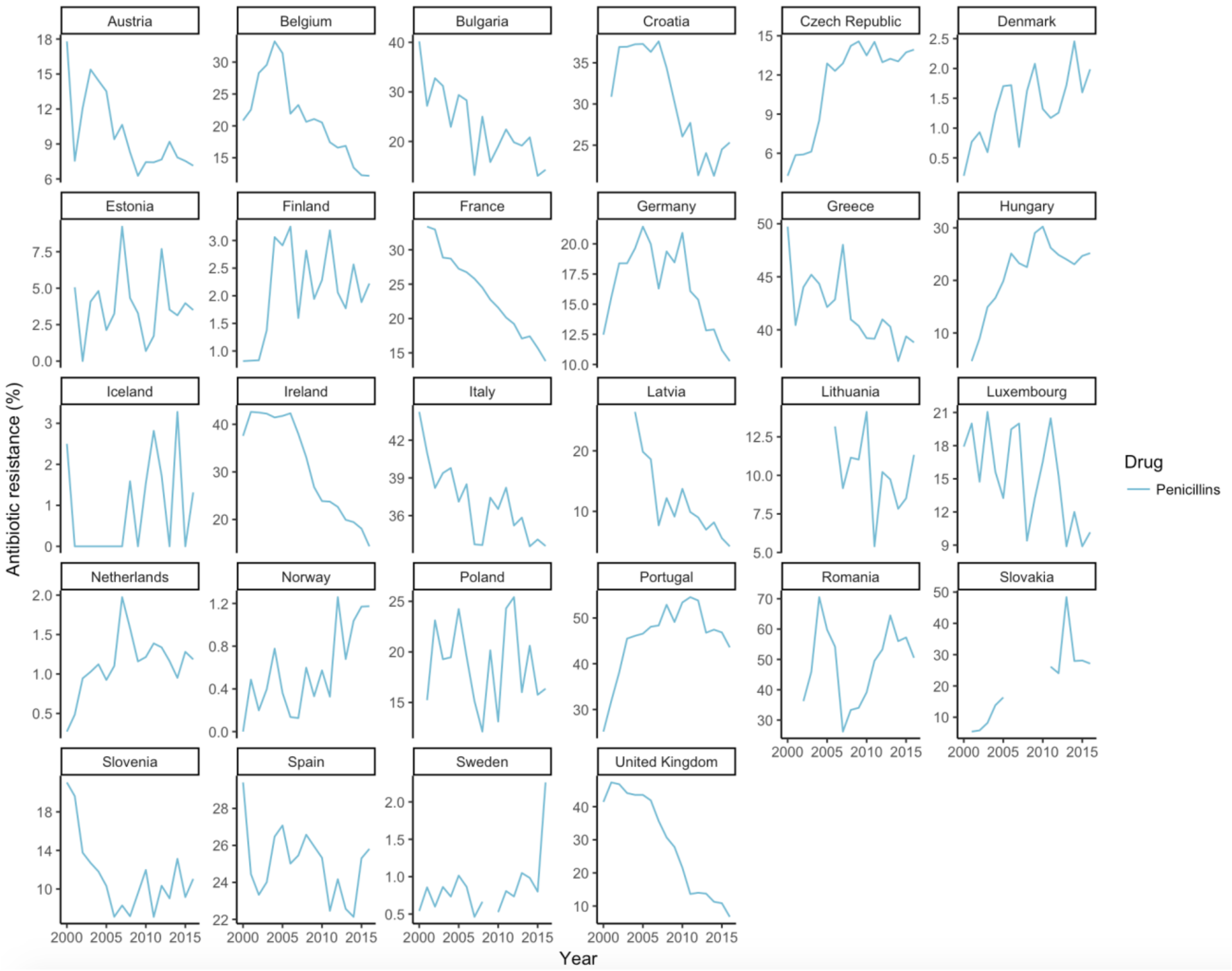

Figure S3. Trends in antibiotic resistance (%) for *S. aureus* and methicillin between 2000-2016 across 28 countries in Europe.

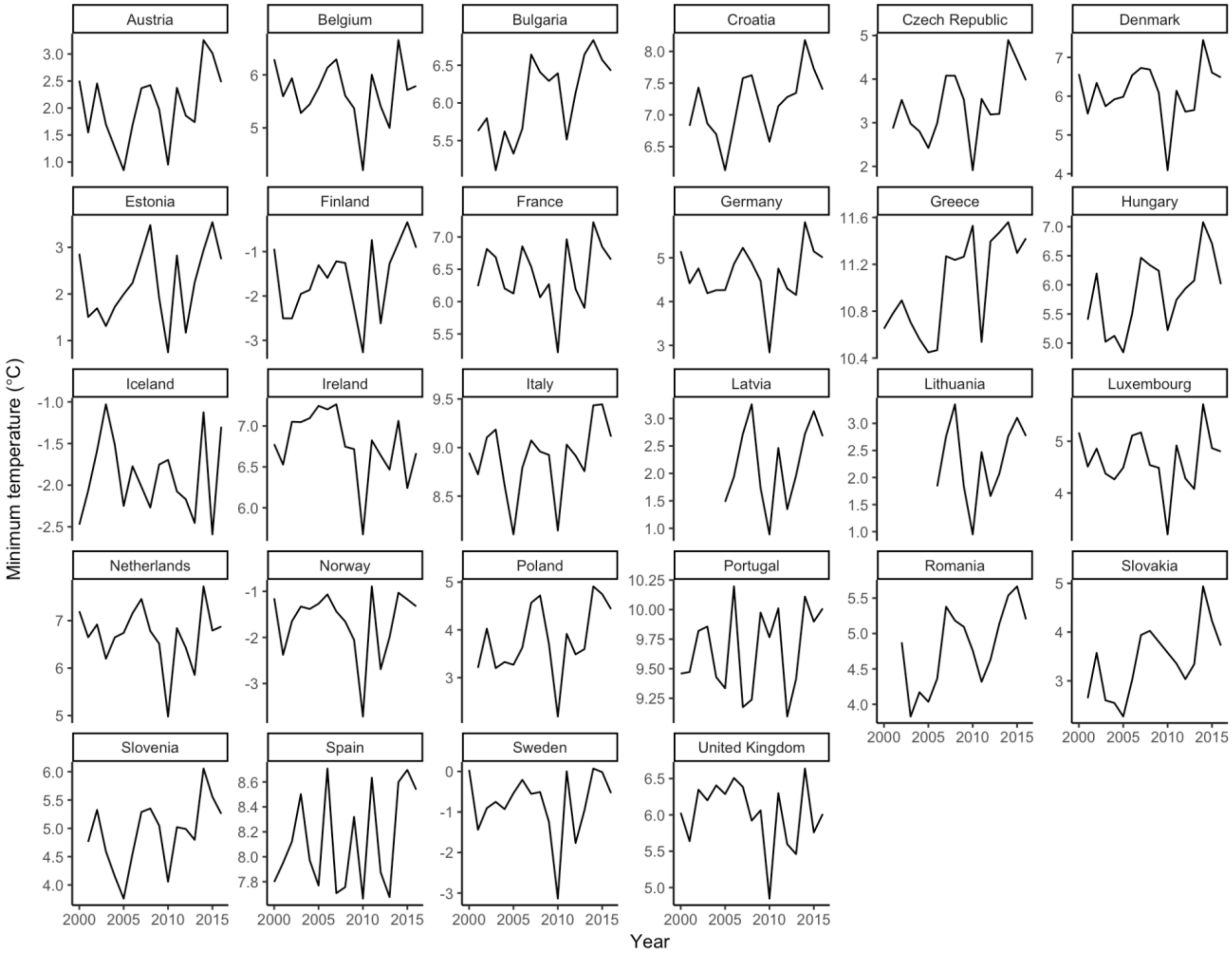

Figure S4. Trends in minimum temperature between 2000-2016 across 28 countries in Europe.

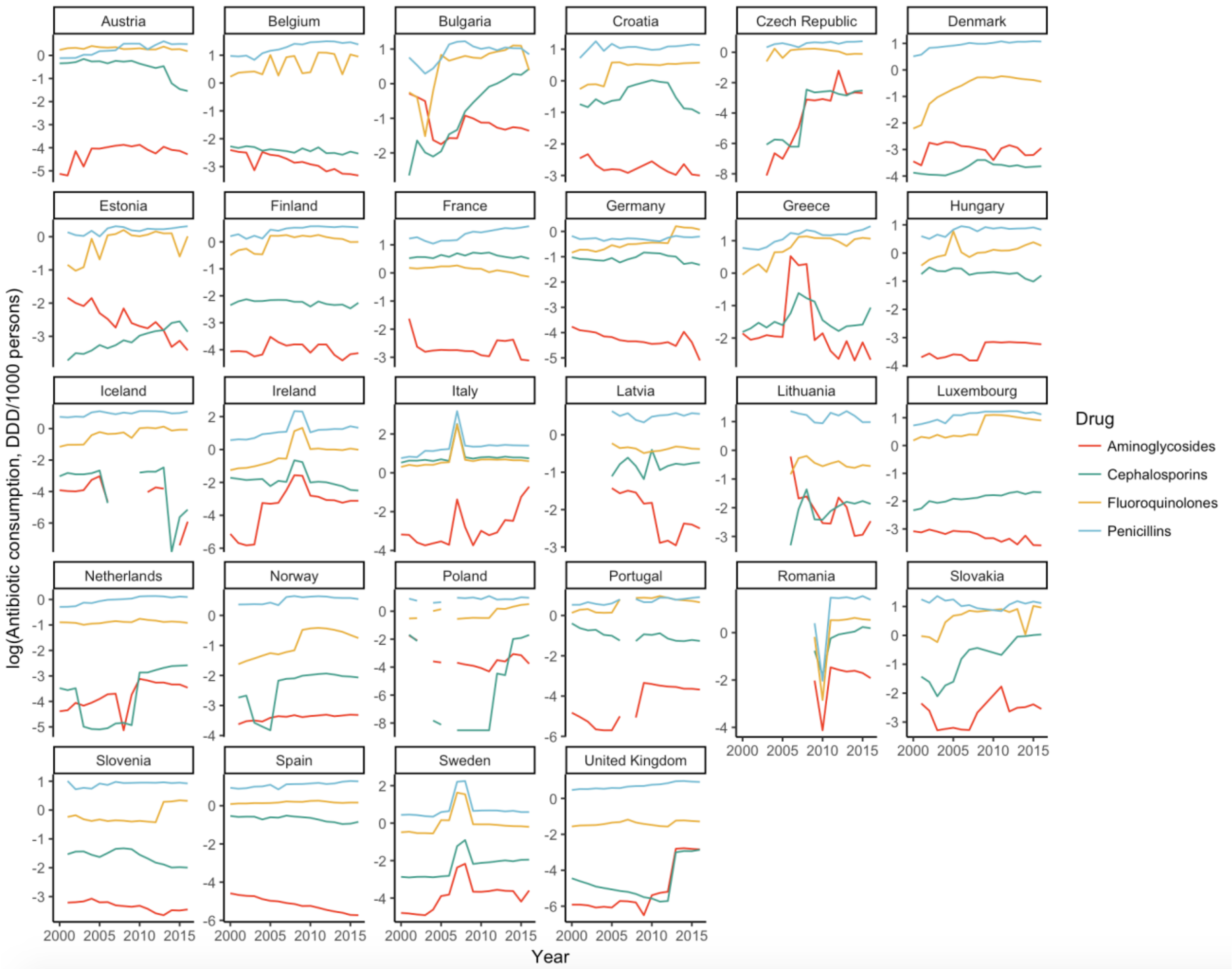

Figure S5. Trends in antibiotic consumption (prescriptions per 1000 inhabitants per day) between 2000-2016 across 28 countries in Europe, for each antibiotic class. Antibiotic consumption is represented on the log scale to visualize large differences in scale between antibiotic classes (colored lines).

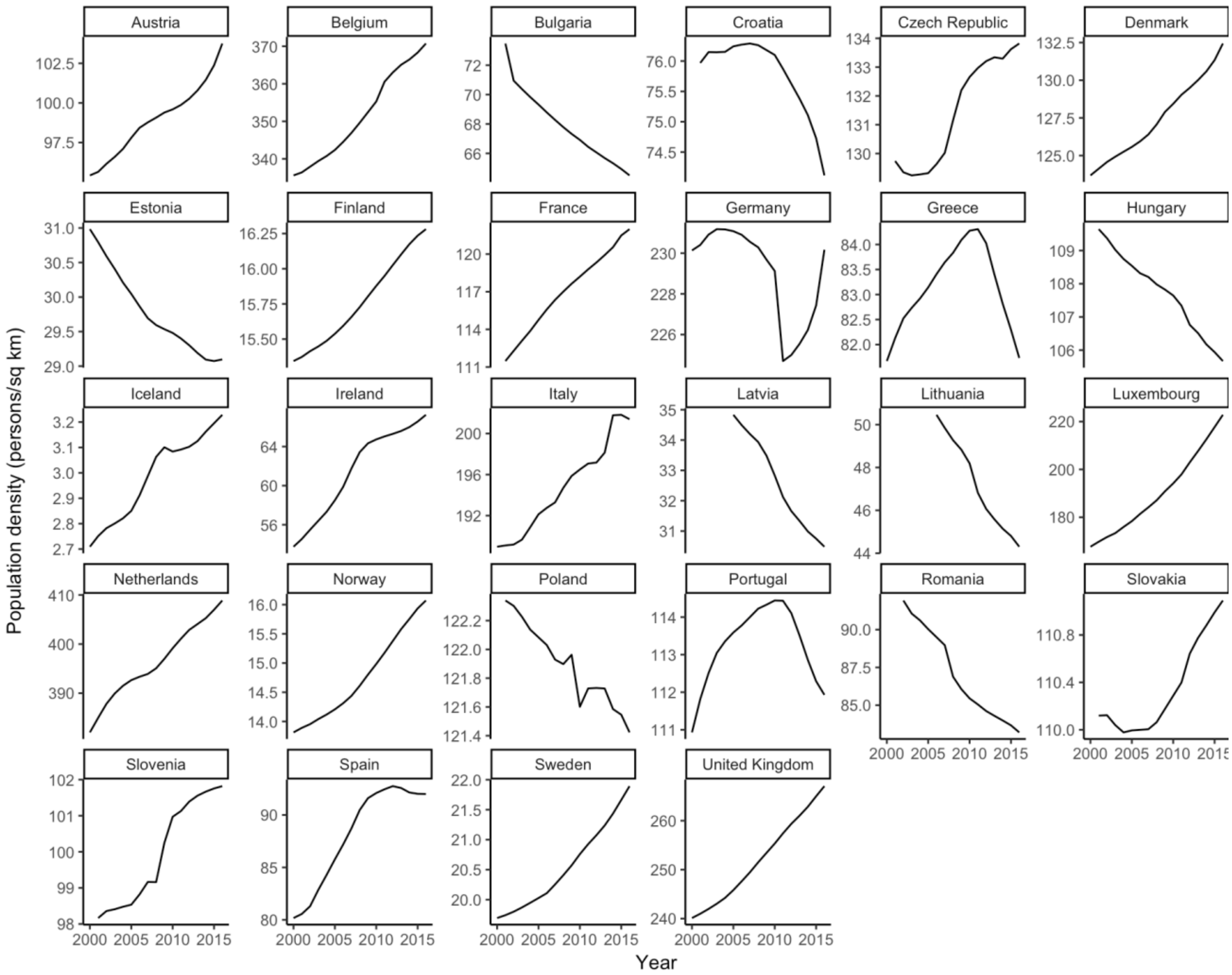

Figure S6. Trends in population density (persons/km²) between 2000-2016 across 28 countries in Europe.

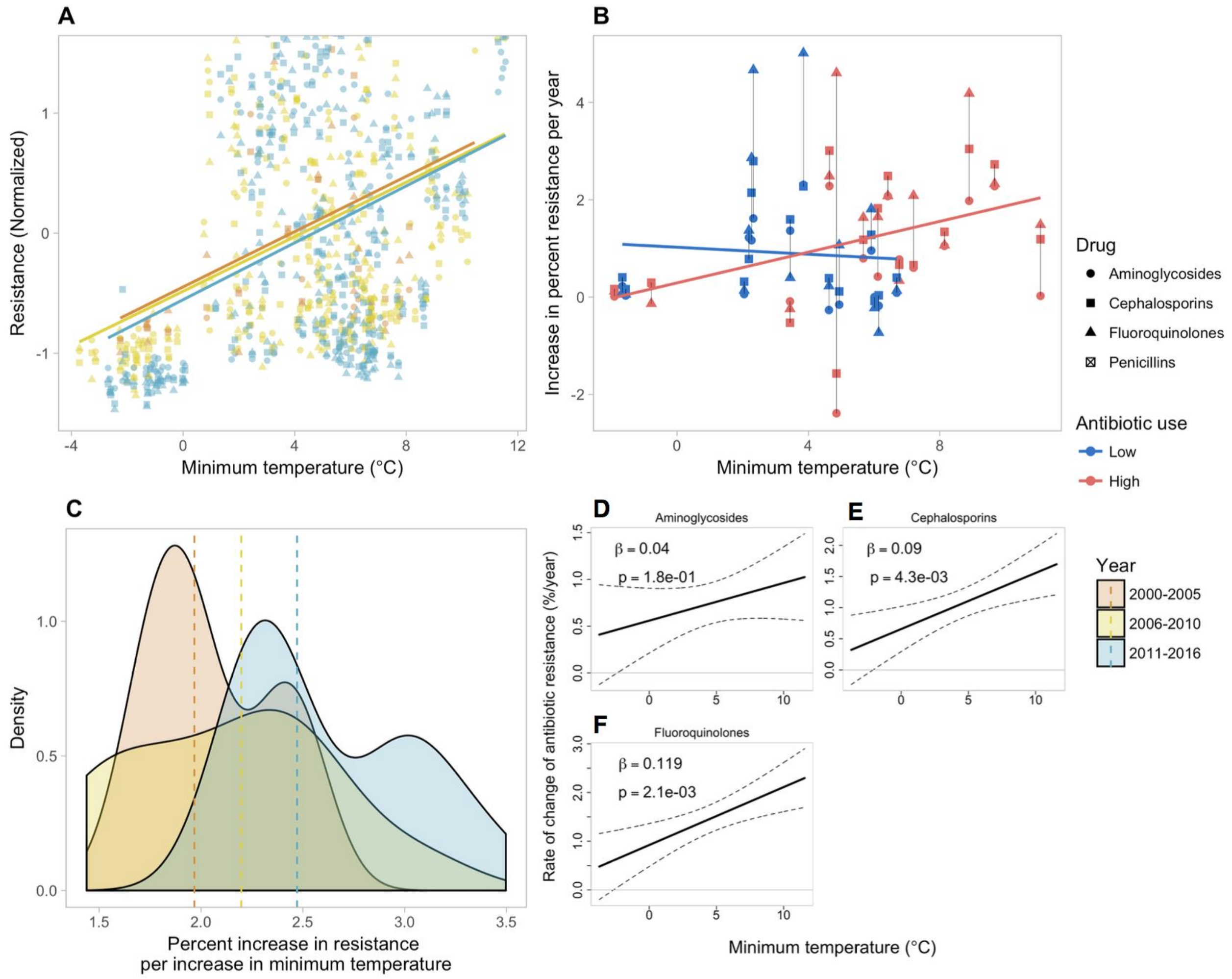

Figure S7. Change in the relationship between antibiotic resistance and minimum temperature over time for *K. pneumoniae*.

(A) Normalized antibiotic resistance versus minimum temperature (°C) for 3 antibiotic classes and all 28 countries, stratified by 5-6 year intervals. (B) Speed of increase of antibiotic resistance versus average minimum temperature, stratified by average median antibiotic consumption (low/high). Each country is represented by a collection of vertical points. (C) Density distributions of association measures (slopes) between antibiotic resistance and minimum temperature, stratified by time and with median densities (by year) marked by vertical dashed lines. (D—F) Change of antibiotic resistance over time as a function of minimum temperature for (D) aminoglycosides, (E) 3rd-generation cephalosporins, and (F) fluoroquinolones, with 95% confidence intervals. Estimates for (D—F) were obtained from multivariable models adjusting for country, minimum temperature (°C), year, population density (persons/km²), antibiotic consumption, and the interaction between year and minimum temperature. Beta coefficients and p-values are given for the interaction between minimum temperature and year, with 95% confidence intervals calculated using the standard error of Equation 3, $\sqrt{Var(b_2) + Tmin^2 \times Var(b_3) + 2Tmin \times Cov(b_2, b_3)}$, where b_2 and b_3 are estimates of β_2 and β_3.

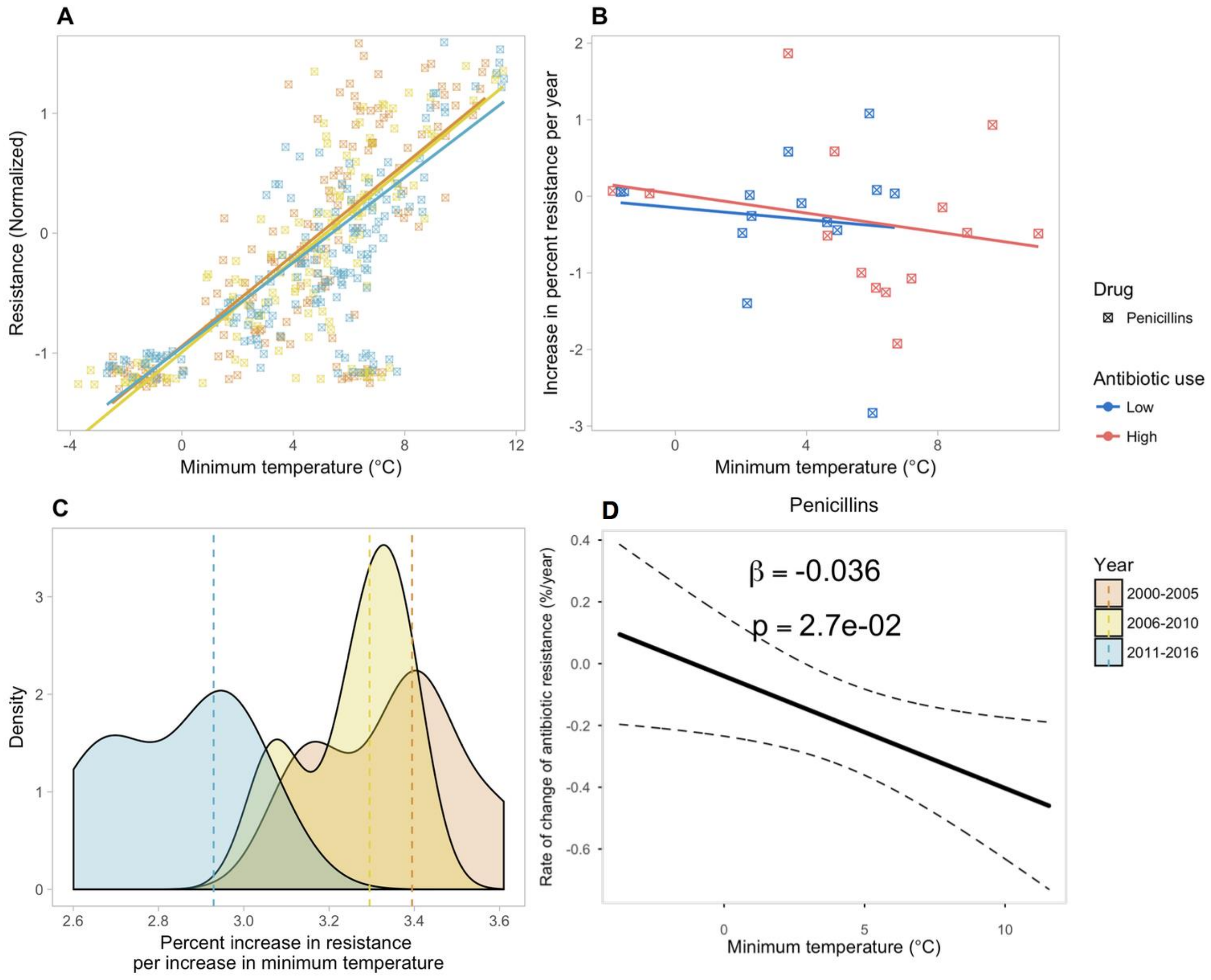

Figure S8. Change in the relationship between antibiotic resistance and minimum temperature over time for *S. aureus*.

(A) Normalized antibiotic resistance versus minimum temperature (°C) for penicillins and all 28 countries, stratified by 5-6 year intervals. (B) Speed of increase of antibiotic resistance versus average minimum temperature, stratified by average median antibiotic consumption (low/high). (C) Density distributions of association measures (slopes) between antibiotic resistance and minimum temperature, stratified by time and with median densities (by year) marked by vertical dashed lines. Each country is represented by a collection of vertical points. (D) Change of antibiotic resistance over time as a function of minimum temperature for methicillin, with 95% confidence intervals. Estimates for (D) were obtained from multivariable models adjusting for country, minimum temperature (°C), year, population density (persons/km²), antibiotic consumption, and the interaction between year and minimum temperature. Beta coefficients and p-values are given for the interaction between minimum temperature and year, with 95% confidence intervals calculated using the standard error of Equation 3,

$\sqrt{Var(b_2) + Tmin^2 \times Var(b_3) + 2Tmin \times Cov(b_2, b_3)}$, where b_2 and b_3 are estimates of β_2 and β_3.

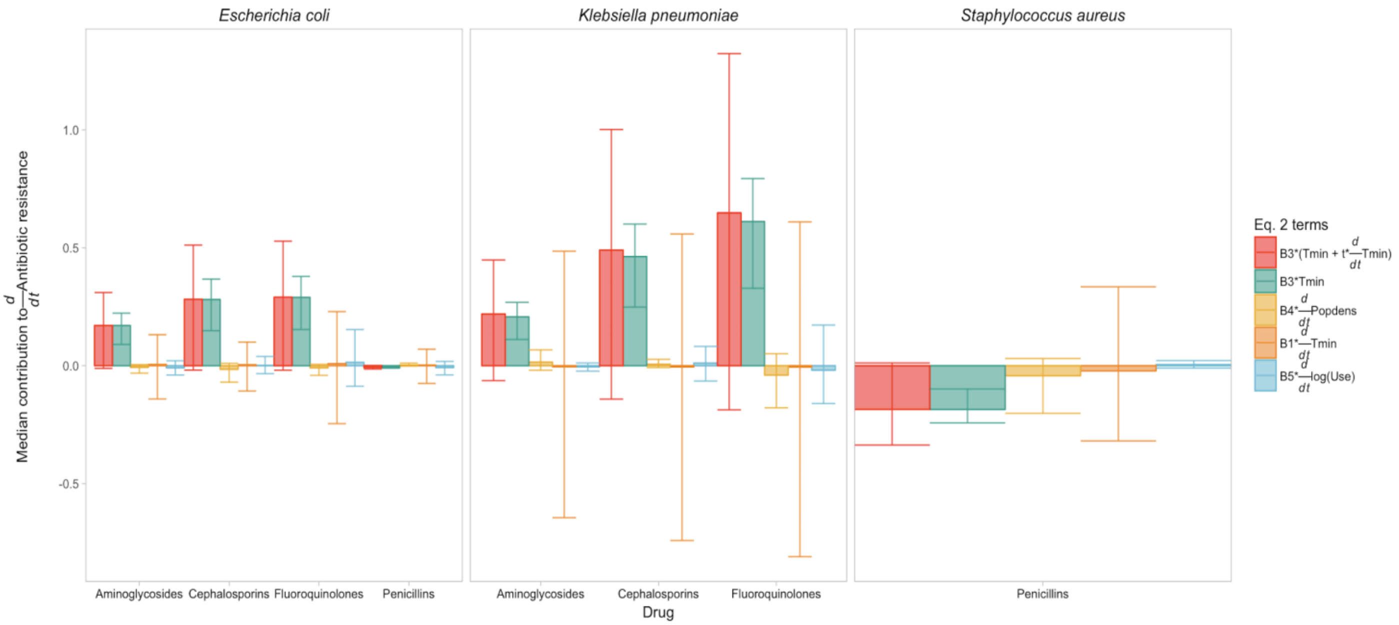

Figure S9. Contribution of Equation 2 terms to the rate of change of antibiotic resistance, by pathogen and antibiotic class. For all countries and years, the contribution of each term to the rate of change of antibiotic resistance was computed by Equation 2, to produce a distribution of values across geographies and time. Here, the median contribution of each term to the rate of change of antibiotic resistance is shown (bar plot), with error bars marking the 25th and 75th percentiles.

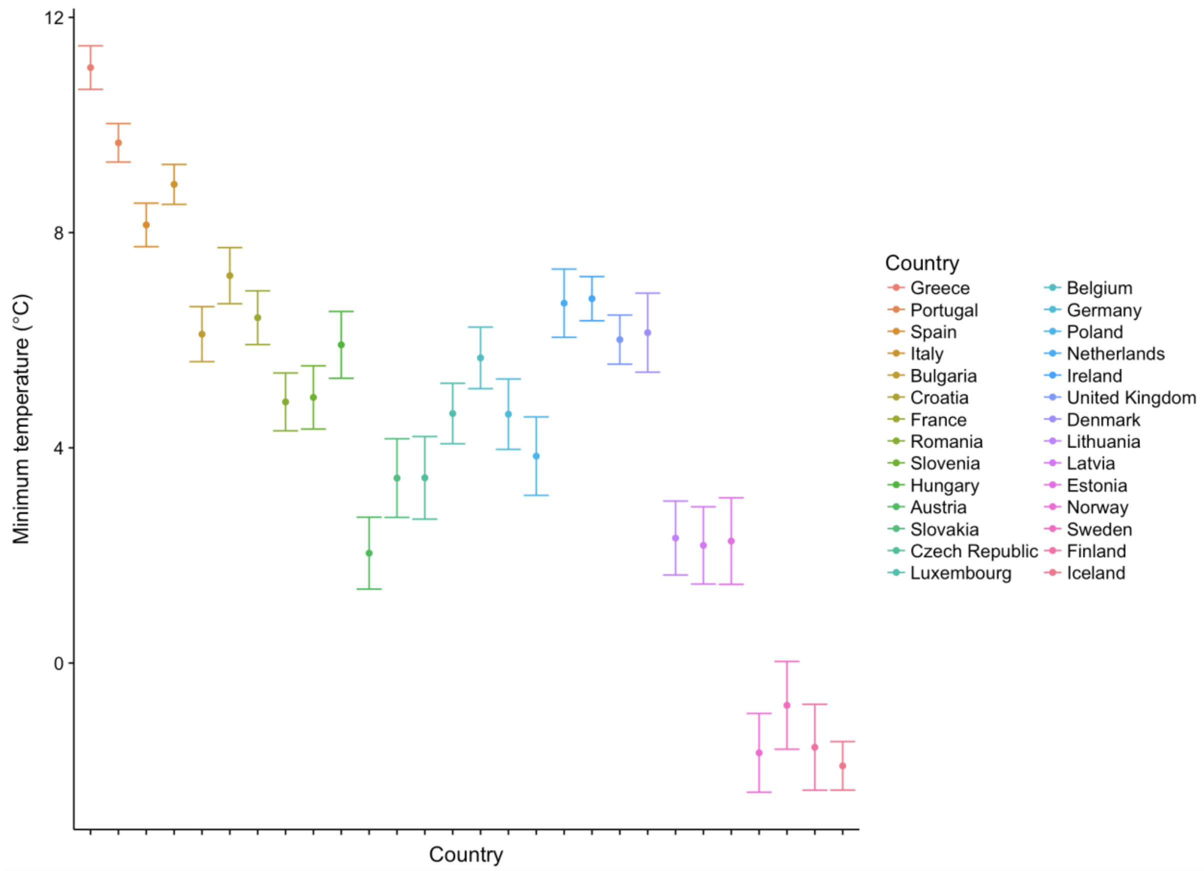

Figure S10. Within- and between-country variability in minimum temperature over 2000-2016. The mean and standard error bars of minimum temperature across 17 years is shown by country. Within-country variability in minimum temperature is smaller than between-country variability in minimum temperature.

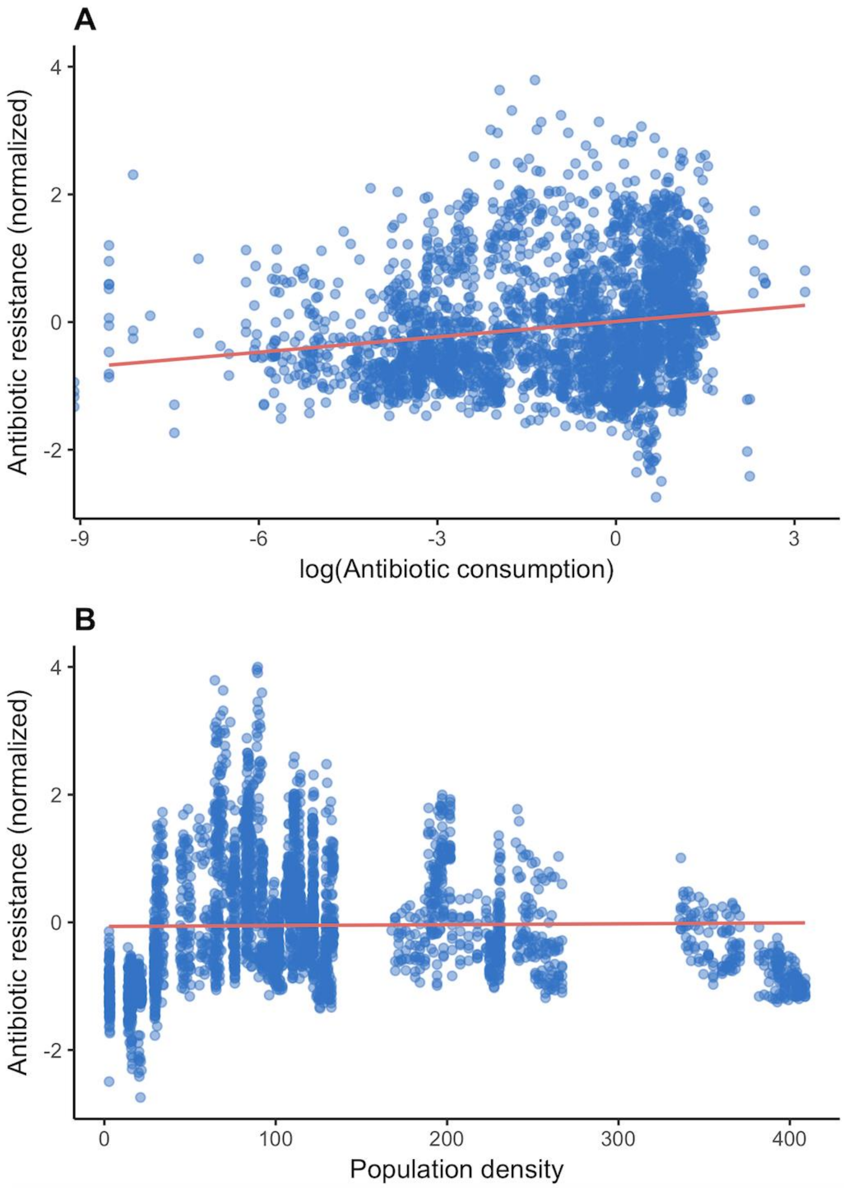

Figure S11. Additional important predictors of antibiotic resistance. Scatter plots of the association between mean normalized antibiotic resistance with (A) log of antibiotic consumption (prescriptions per 1000 inhabitants per day) and (B) population density (persons/km^2), across *E. coli*, *K. pneumoniae*, and *S. aureus*. Linear trend lines are estimated in red.

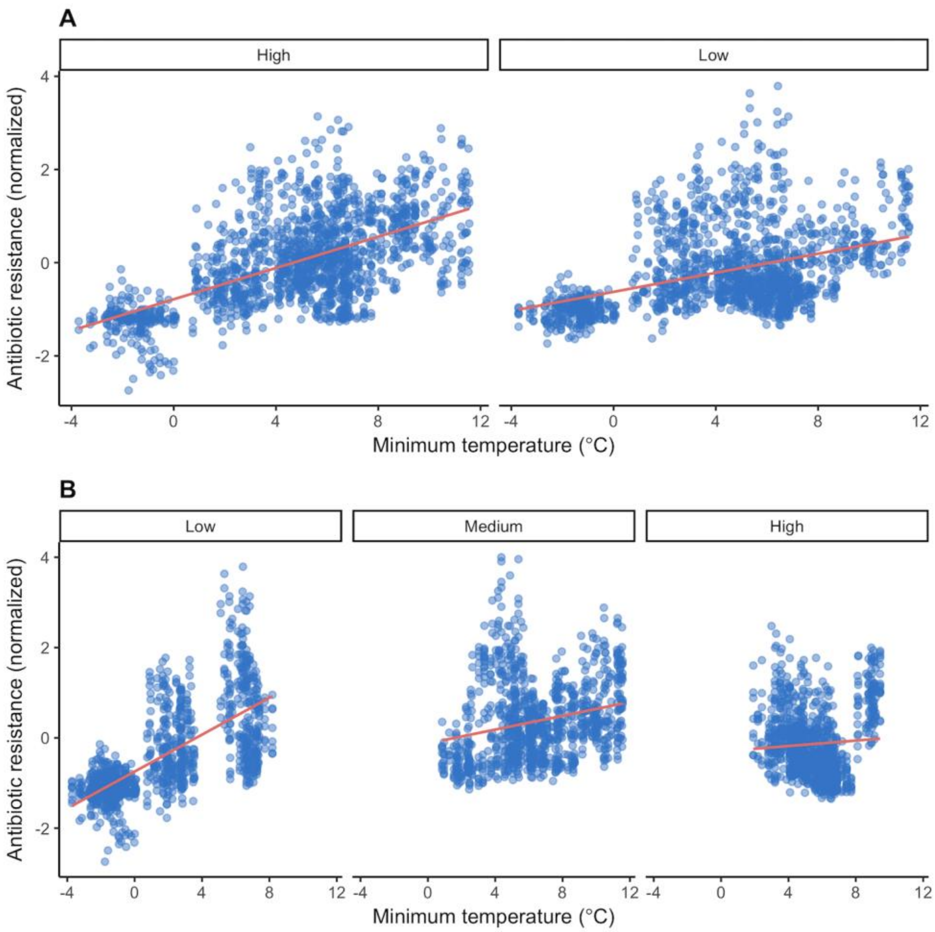

Figure S12. Assessing the relationship of minimum temperature and antibiotic resistance across levels of other covariates. Scatter plots of normalized antibiotic resistance versus minimum temperature (ºC) by: (A) median of antibiotic consumption (prescriptions per 1000 inhabitants per day) and (B) tertile of population density (persons/km^2). Data from all pathogens (*E. coli*, *K. pneumoniae*, and *S. aureus*) are represented. Linear trend lines are estimated in red.

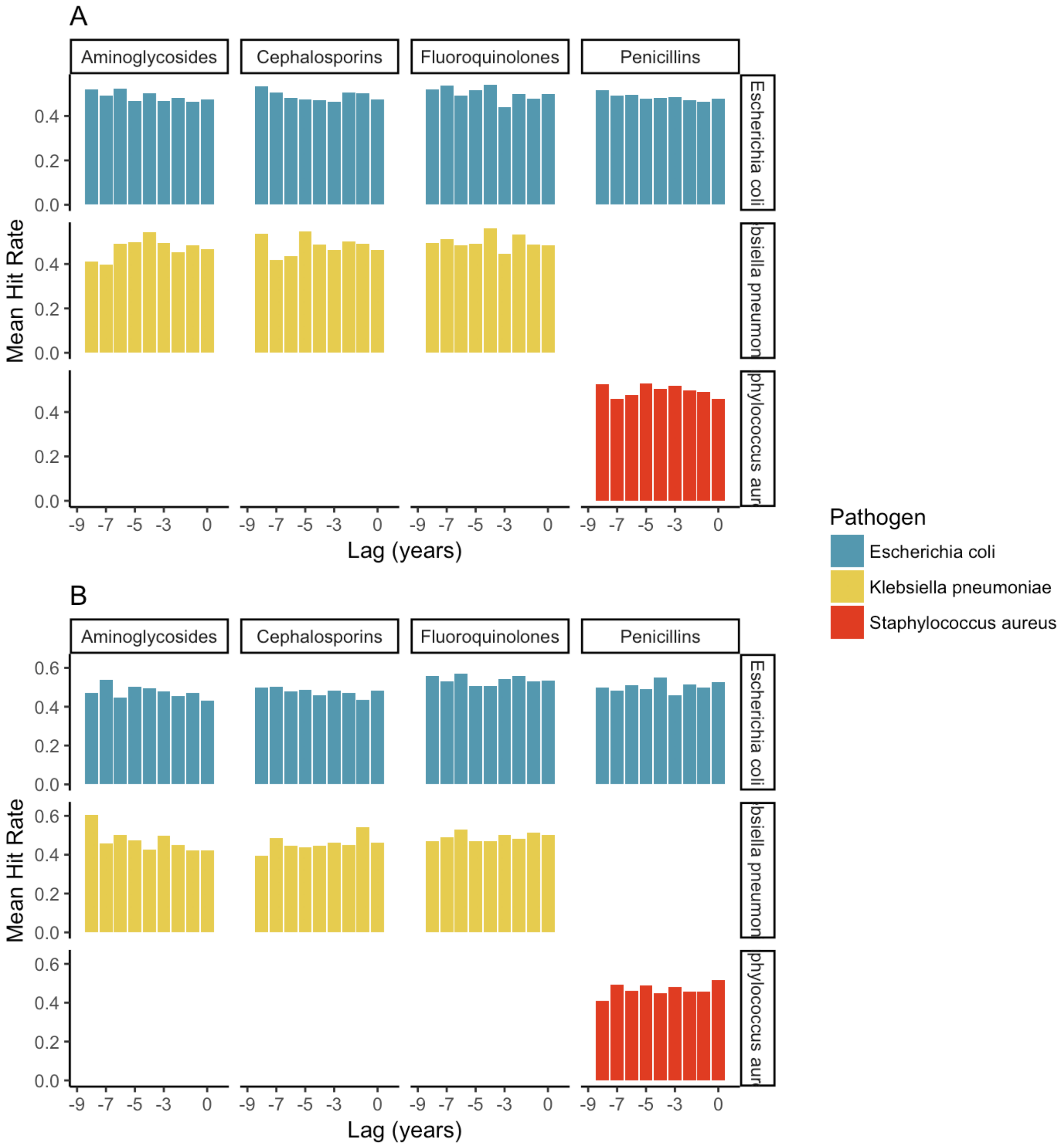

Figure S13. Mean hit rates capturing the congruence of directional changes in antibiotic resistance and (A) minimum temperature and (B) antibiotic consumption across 28 countries, at temporal lags of between 0 and 9 years, for each pathogen and antibiotic class. The hit rate captures the proportion of years for which the annual changes in antibiotic resistance and predictors are in the same direction, and was computed by country, pathogen, and antibiotic class for minimum temperature lags of between 0 and 9 years. Here, the mean hit rate across countries is shown.

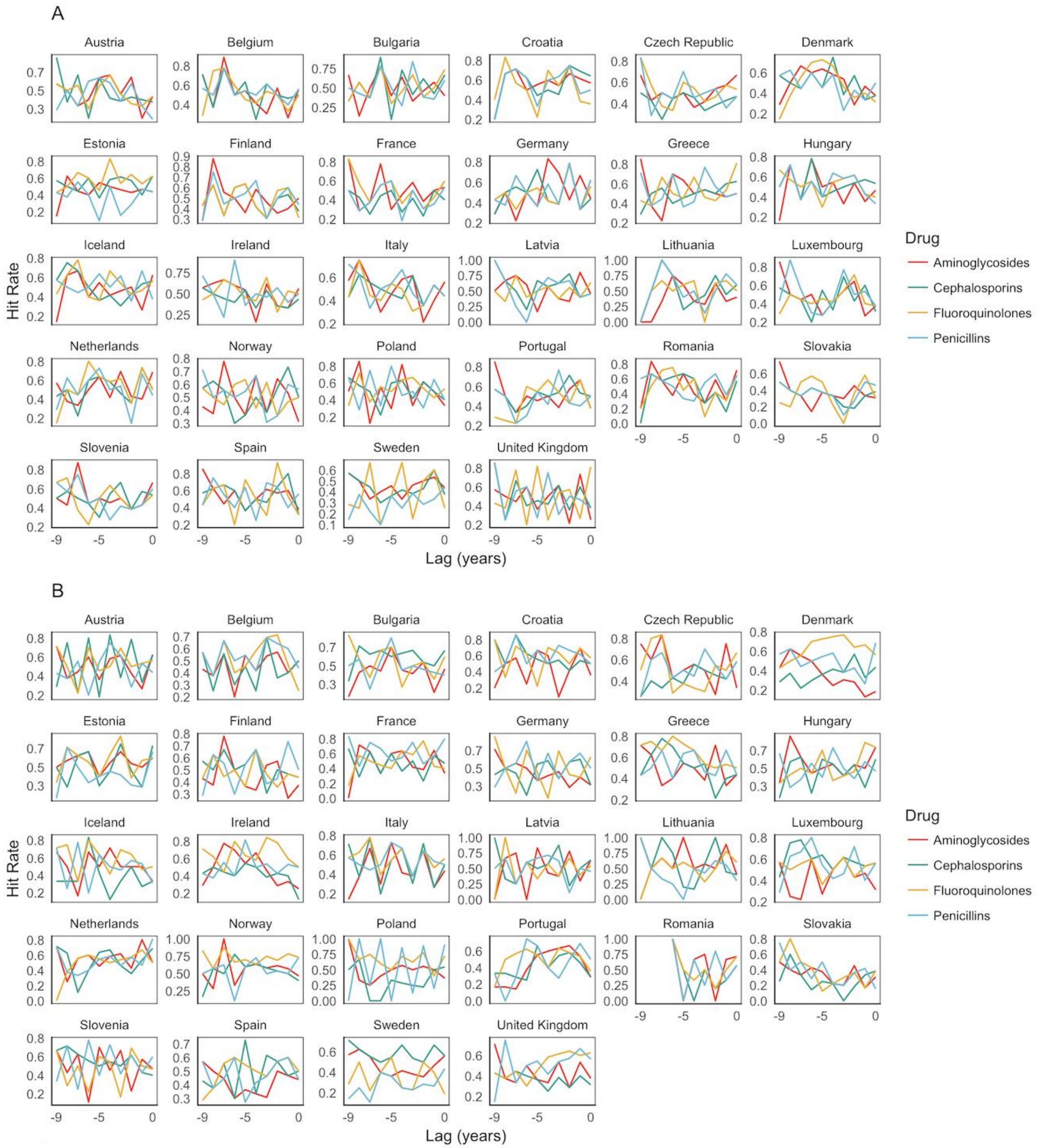

Figure S14. *E. coli* hit rates measuring the congruence of directional changes in antibiotic resistance and (A) minimum temperature and (B) antibiotic consumption, for multiple lags by country and antibiotic class. The hit rate captures the proportion of years for which the annual changes in antibiotic resistance and predictors are in the same direction, and was computed for temperature lags of between 0 and 9 years. Minimum temperature and antibiotic consumption precede antibiotic resistance at negative lags.

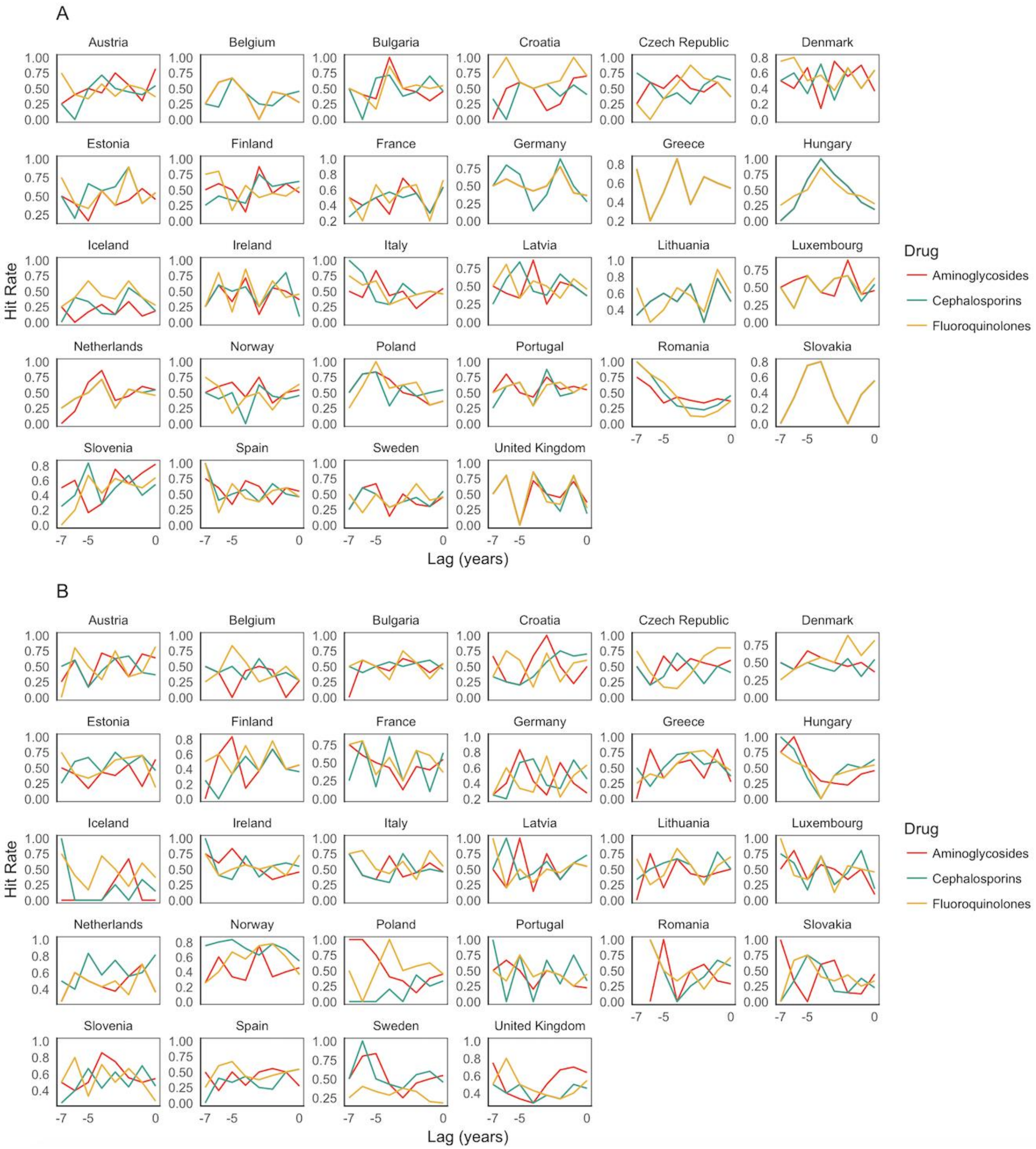

Figure S15. *K. pneumoniae* hit rates measuring the congruence of directional changes in antibiotic resistance and (A) minimum temperature and (B) antibiotic consumption, for multiple lags by country and antibiotic class. The hit rate captures the proportion of years for which the annual changes in antibiotic resistance and predictors are in the same direction, and was computed for temperature lags of between 0 and 7 years (due to shorter availability of *K. pneumoniae* resistance data). Minimum temperature and antibiotic consumption precede antibiotic resistance at negative lags.

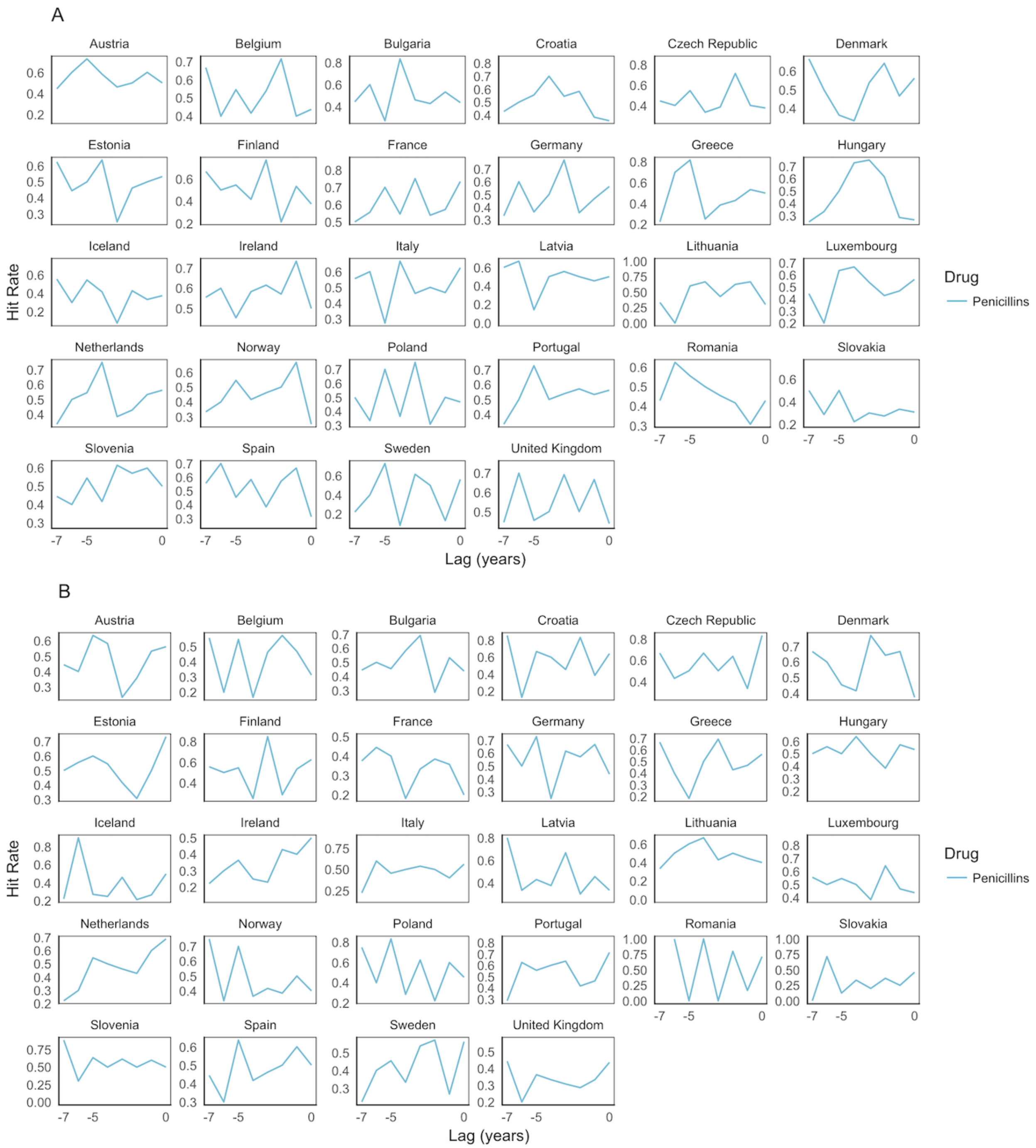

Figure S16. *S. aureus* hit rates measuring the congruence of directional changes in antibiotic resistance and (A) minimum temperature and (B) antibiotic consumption, for multiple lags by country and antibiotic class. The hit rate captures the proportion of years for which the annual changes in antibiotic resistance and predictors are in the same direction, and was computed for temperature lags of between 0 and 9 years. Minimum temperature and antibiotic consumption precede antibiotic resistance at negative lags.
